# Cytosolic condensates rich in polyserine define subcellular sites of tau aggregation

**DOI:** 10.1073/pnas.2217759120

**Published:** 2023-01-10

**Authors:** Evan Lester, Meaghan Van Alstyne, Kathleen L. McCann, Spoorthy Reddy, Li Yi Cheng, Jeff Kuo, James Pratt, Roy Parker

**Affiliations:** ^a^Medical Scientist Training Program, University of Colorado Anschutz Medical Campus, Aurora, CO 80045; ^b^Department of Biochemistry, University of Colorado, Boulder, CO 80303; ^c^HHMI, University of Colorado, Boulder, CO 80303

**Keywords:** tau, SRRM2, Alzheimer’s disease, frontotemporal dementia, RNA-binding proteins

## Abstract

Tau aggregates are a hallmark of multiple neurodegenerative diseases and can contain RNAs and RNA-binding proteins, including serine/arginine repetitive matrix protein 2 (SRRM2) and pinin (PNN). However, how these nuclear proteins mislocalize and their influence on the prion-like propagation of tau aggregates is unknown. We demonstrate that polyserine repeats in SRRM2 and PNN are necessary and sufficient for recruitment to tau aggregates. Moreover, we show tau aggregates preferentially grow in association with endogenous cytoplasmic assemblies—mitotic interchromatin granules and cytoplasmic speckles (CSs)—which contain SRRM2 and PNN. Polyserine overexpression in cells nucleates assemblies that are sites of tau aggregate growth. Further, modulating the levels of polyserine-containing proteins results in a corresponding change in tau aggregation. These findings define a specific protein motif, and cellular condensates, that promote tau aggregate propagation. As CSs form in induced pluripotent stem cell (iPSC) derived neurons under inflammatory or hyperosmolar stress, they may affect tau aggregate propagation in neurodegenerative disease.

## Significance

Tau aggregates are a pathologic hallmark of several neurodegenerative diseases including Alzheimer’s disease and forms of frontotemporal dementia. Despite their ubiquitous presence in pathology, little is known about how they form in cells. Here, we show that specific biological condensates referred to as mitotic interchromatin granules (MIGs) and related cytoplasmic speckles (CSs) serve as preferred sites of tau aggregate growth in cells. We also show that polyserine repeats in MIG/CS resident RNA binding proteins are responsible for their interaction with tau. Further, altering the level of polyserine in cells leads to a corresponding change in tau aggregation in a model system. This work provides a mechanistic understanding of the origin of tau aggregates.

Tau inclusions are a pathological hallmark defining over 20 neurodegenerative diseases including Alzheimer’s disease (AD), corticobasal degeneration (CBD), and hereditary frontotemporal dementia with parkinsonism-17 (FTDP-17), collectively classified as tauopathies (1). While loss-of-function mechanisms can contribute to disease, tau knockout mice have no overt neurodegenerative phenotype and show only mild deficits at advanced ages (2). Instead, several observations argue that the formation and spread of tau oligomers or aggregates can cause neurodegenerative disease. For example, tau mutations in familial FTDP-17 promote tau aggregation (3), induction of tau aggregates leads to toxicity in cells (4), and targeted reduction of tau and inhibition of tau aggregation reverses cognitive impairments in tauopathy mouse models (5–7). The aggregation of tau has been proposed to propagate through prion-like mechanisms originating via misfolding of an initial seed and progressing into larger, fibrillar inclusions (8). Nonetheless, the mechanistic basis for tau-mediated aggregation and neurodegeneration remains incompletely understood.

A key area of interest in the formation of tau fibers and aggregates are the molecules and subcellular locations that modulate tau aggregation formation and propagation. Previous work has shown that tau binds microtubules and pathogenic mutations or post-translational modifications reduce this association leading to increased tau aggregate formation (9). Yet, in vitro tau fibrillization typically requires a polyanionic co-factor such as RNA or heparin (10–12). This in vitro requirement suggests that tau aggregation in cells will require cofactors, which remain unknown. Interestingly, tau can form assemblies in vitro with RNA (13) suggesting an RNA-containing condensate within cells might contribute to tau aggregate formation and/or propagation, although direct evidence for a specific cellular condensate that could promote tau aggregation is lacking.

We recently showed that nuclear tau aggregates—which can be observed in cell lines, mouse models, or human post-mortem samples—are observed in association with nuclear speckles (14, 15). Nuclear speckles are a condensate made up of nascent transcripts and components of the transcription or RNA processing machinery (16). Nuclear tau aggregates in model systems disrupt the organization, dynamics, and composition of nuclear speckles and this could contribute to the neurotoxicity and RNA splicing defects seen in some tauopathies (14). Moreover, we observed the mislocalization of some nuclear speckle proteins to cytosolic tau aggregates (14). We found that two of the most prominently mislocalized proteins are PNN and SRRM2. The mislocalization of SRRM2 to cytoplasmic tau aggregates occurs in both tau mouse models of disease and tauopathy patient brains (14, 17). SRRM2 is an RNA binding protein (RBP) involved in RNA splicing and RNA sequencing shows tau aggregation induces RNA splicing changes in several model systems (14, 18), as well as in patients with AD (19, 20). These features liken tauopathies to other neurodegenerative diseases in which dysfunction and mislocalization of RBPs leads to dysregulated RNA processing and gene expression (21). However, how the interaction between nuclear speckle components and tau aggregates contributes to tau aggregate formation and/or toxicity remains unknown.

Here, we investigated the mechanisms mediating the mislocalization of nuclear speckle proteins to tau inclusions and their involvement in tau aggregation. We identified polyserine stretches in SRRM2 and PNN that contribute to, and are sufficient for, recruitment to tau aggregates. We also identified cytoplasmic condensates containing SRRM2, referred to as mitotic interchromatin granules (MIGs) and cytoplasmic speckles (CSs), that serve as preferential sites of tau aggregate growth. Importantly, the reduction of PNN or overexpression of polyserine domains correspondingly decreases or increases tau aggregation. These findings delineate homopolymeric serine stretches as mediators of protein recruitment to tau aggregates as well as a defining feature of assemblies that specify a preferred subcellular location for tau aggregation.

## Results

### The C-Terminal Regions of SRRM2 and PNN Mediate Association With Tau Aggregates.

**Fig. 1.**
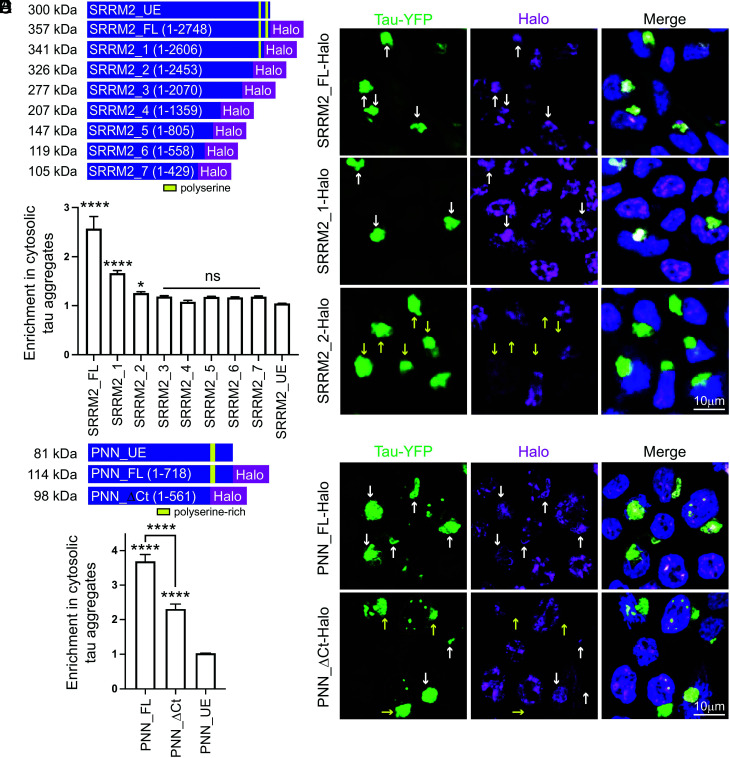
SRRM2 and PNN C-termini mediate enrichment in tau aggregates. (*A*) Schematic of SRRM2 truncations generated through CRISPaint in HEK293 tau biosensor cells where Halo tags were introduced into endogenous SRRM2 loci followed by a polyadenylation signal to create tagged and truncated proteins. Yellow regions denote two polyserine stretches in the SRRM2 C-terminus (*B*) Immunofluorescence of tau-YFP (green), Halo (magenta), and DAPI (blue) in SRRM2_FL-Halo, SRRM2_1-Halo, and SRRM2_2-Halo lines showing enrichment of SRRM2 (white arrows) or lack of enrichment (yellow arrows) in tau aggregates seeded via lipofection of clarified brain homogenate from tau transgenic mice (Tg2541). Images of all truncations can be found in *SI Appendix*, Fig. S2*A*. (*C*) Quantification of the ratio of Halo mean intensity within tau aggregates relative to Halo mean intensity in the cytoplasm for SRRM2 truncations. n = 75 images quantified from three biological replicates per group. Statistics performed with one-way ANOVA with comparison to SRRM2_UE. (*D*) Schematic of PNN truncation made through CRISPaint introducing a Halo tag at the endogenous PNN loci. (*E*) Representative images of Halo labeling (magenta) and tau-YFP (green) in full-length Halo tagged PNN (PNN_FL-Halo) and C-terminal truncated PNN (PNN_DCt-Halo) cell lines showing enrichment of PNN (white arrows) or no enrichment (yellow arrow) in tau aggregates. (*F*) Quantification of unedited (PNN_UE), PNN_FL-Halo, and PNN_DCt-Halo enrichment in cytoplasmic tau aggregates as in (*C*). n > 788 cells from three biological replicates per group. For all plots, data represent mean and 95% CI. Statistics performed with one-way ANOVA. (*) *P* < 0.05 (****) *P* < 0.0001.

Our previous work showed the long C-terminal disordered region of SRRM2 was required for its mislocalization to cytoplasmic tau aggregates (14). To determine whether there is a specific motif that dictates SRRM2-tau interactions, we used HEK293 tau biosensor cells to generate a series of cell lines with truncated SRRM2 by inserting Halo tags into chromosomal copies of the SRRM2 gene using the CRISPaint system (22, 23). The HEK293 tau biosensor cells express both cyan fluorescent protein (CFP) and yellow fluorescent protein (YFP) tagged forms of the tau K18 fragment with the P301S mutation and form bright FRET+ (Förster resonance energy transfer) tau aggregates upon lipofection of exogenous tau seeds isolated from the brains of Tg2541 (P301S) tauopathy mice (24). All endogenous Halo-tagged SRRM2 truncations were expressed well, conjugated to JF646 fluorophores, and were detectable at the appropriate size (Fig. 1*A*, and *SI Appendix*, Figs. S1*A* and S2*A*). The multiple bands observed in truncations 5 to 7 could represent unannotated splice isoforms of SRRM2 or post-translational modifications, which are revealed because of the smaller size of these truncation proteins (*SI Appendix*, Fig. S1*A*).

This analysis demonstrated that the last 294 amino acids in the C-terminal region of SRRM2 are necessary for recruitment to tau aggregates. Specifically, we observed deletion of the last 146 amino acids (SRRM2_1, a.a. 2606 to 2752) significantly reduced enrichment in tau aggregates (from a mean enrichment of 2.57 for full-length SRRM2 to 1.67) while a larger 294 amino acid deletion (SRRM2_2, a.a. 2458 to 2752) led to a reduction in enrichment to a small yet still statistically significant level relative to untagged SRRM2 (mean enrichment of 1.26) (Fig. 1 *B* and *C*). All larger deletions failed to show statistically significant enrichment in tau aggregates (Fig. 1 *B* and *C* and *SI Appendix*, Fig. S2*A*). Furthermore, SRRM2 truncations 6 and 7 display diffuse nuclear localization indicating amino acids 558 to 805 are required for normal recruitment of SRRM2 to nuclear speckles (*SI Appendix*, Fig. S2*A*).

SRRM2 has two homopolymeric serine stretches in the C-terminal 294 amino acids, one that is 42 residues and a second that is 25 residues (Fig. 1*A*, yellow boxes). The SRRM2_1 truncation removes the 42-polyserine stretch while the SRRM2_2 truncation removes both the 42 and 25-polyserine stretches. These results suggested two regions within the C-terminus of SRRM2—each containing a polyserine domain—are responsible for most of the enrichment of SRRM2 in tau aggregates.

The nuclear speckle protein PNN also mislocalizes to cytoplasmic tau aggregates (*SI Appendix*, Fig. S1*C*) (14). The C-terminus of PNN contains a region of 66 amino acids of which 50 (76%) are serine residues. To determine whether this serine-rich region of PNN mediates associations with tau aggregates, we utilized CRISPaint to generate cell lines expressing Halo-tagged full-length PNN (PNN_FL) or a truncated (PNN_Ct) form with deletion of the terminal 157 amino acids including the serine-rich region (Fig. 1*D* and *SI Appendix*, Fig. S1*B*). We observed the C-terminal truncation of PNN resulted in a significant reduction in recruitment to tau aggregates compared to the full-length protein (Fig. 1 *E* and *F*), providing additional evidence that serine-rich regions mediate associations with tau aggregates.

While the polyserine-rich regions of SRRM2 and PNN appear to be responsible for most of the enrichment into tau aggregates, there remains residual enrichment in constructs lacking polyserine domains (i.e., SRRM2_2 and PNN_ΔCt) (Fig. 1 *C* and *F*). It is possible that other regions of these proteins can also facilitate recruitment to tau aggregates via direct interactions or via intermediaries. As such, PNN and SRRM2 have been shown to bind one another, and it is possible that SRRM2 could recruit PNN and vice versa through a different domain.

Taken together, these findings identify polyserine or serine-rich domains as elements resulting in mislocalization and recruitment of two nuclear speckle proteins to tau aggregates. Interestingly, there are fewer than 20 human proteins that contain pure stretches of serine longer than 20 amino acids and several of the top serine-repeat-containing proteins are involved in RNA homeostasis (*SI Appendix*, Table S1). In addition to SRRM2 and PNN, we tested SETD1A—a histone methyltransferase implicated in neurodevelopmental disorders that contains 24 consecutive serines—and observed enrichment in tau aggregates (*SI Appendix*, Fig. S1*D*).

### Serine-Rich Protein Domains, or Polyserine Alone, Are Sufficient For Association With Tau Aggregates.

**Fig. 2.**
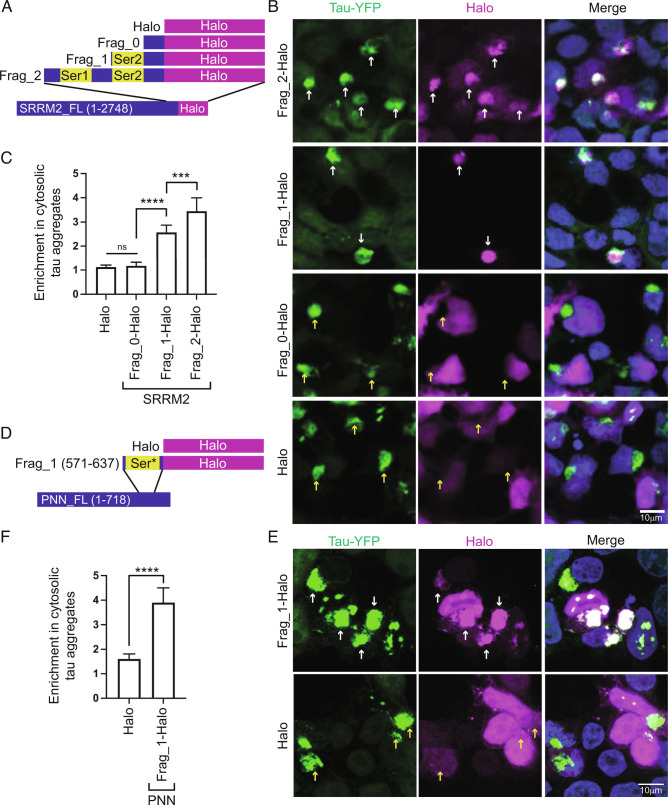
SRRM2 and PNN C-termini are sufficient for localization to tau aggregates. (*A*) Schematic of Halo tagged SRRM2 C-terminal fragment constructs and control. (*B*) Immunofluorescence of tau-YFP (green), Halo (magenta), and DAPI (blue) in HEK293 biosensor cells transfected with constructs in (*A*) and seeded with clarified brain homogenate showing colocalization with tau aggregates (white arrows) and lack of colocalization (yellow arrows). (*C*) Quantification of the ratio of mean intensity of Halo within cytoplasmic tau aggregates relative to Halo signal in the surrounding cytoplasm for SRRM2 C-terminal fragments. n = 25 tau aggregates per group. (*D*) Schematic of construct encoding a Halo tagged serine-rich fragment of PNN. (*E*) Immunofluorescence of tau-YFP (green), Halo (magenta), and DAPI (blue) in HEK293 biosensor cells transfected with constructs in (*D*) and seeded with clarified brain homogenate showing colocalization with tau aggregates (white arrows) and lack of colocalization (yellow arrows). (*F*) Quantification of the ratio of mean intensity of Halo within cytoplasmic tau aggregates relative to Halo signal in the remaining cytoplasm for PNN C-terminal fragment. n > 140 cells from three biological replicates per group. Data shows mean and 95% CI. Statistics performed with one-way ANOVA. (***) *P* < 0.001; (****) *P* < 0.0001.

To determine whether the polyserine-containing regions of SRRM2 and PNN are sufficient for recruitment to tau aggregates, we exogenously expressed Halo-tagged C-terminal SRRM2 fragments containing both polyserine regions (Frag_2: a.a 2458 to 2752), one polyserine region (Frag_1: a.a. 2606 to 2752), or no polyserine regions (Frag_0: a.a. 2651 to 2752) (Fig. 2*A*). We also expressed amino acids 561 to 637 from PNN fused to Halo, where 50 out of 66 amino acids are serine residues (Fig. 2*D*).

We observed that these protein fragments were sufficient to target proteins to tau aggregates proportionate to their serine content. Specifically, Frag_2, with two polyserine domains, accumulated robustly in cytoplasmic tau aggregates; Frag_1, with one polyserine domain, accumulated to a lesser extent; and neither Frag_0 nor Halo alone, which lack polyserine domains, were enriched in tau aggregates (Fig. 2 *B* and *C*). Similarly, the serine-rich region of PNN was sufficient to target Halo to tau aggregates (Fig. 2 *E* and *F*).

To determine whether polyserine itself is sufficient for localization to tau aggregates, we expressed and verified Halo-tagged polyserine repeats of varying lengths (42, 20, 10, 5) (*SI Appendix*, Fig. S2*B*). We then monitored their recruitment to tau aggregates in HEK293 biosensor cells.

**Fig. 3.**
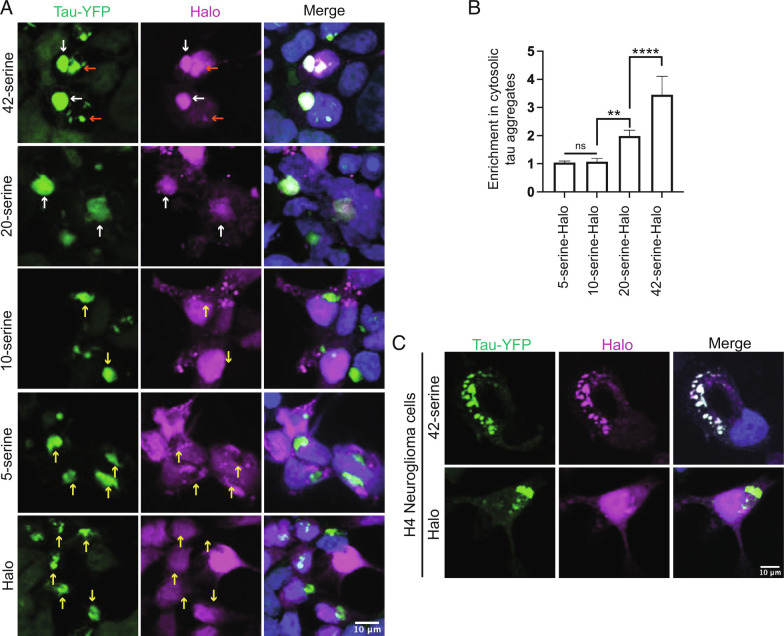
Serine repeats are sufficient for enrichment in tau aggregates. (*A*) Immunofluorescence of tau-YFP (green), Halo (magenta), and DAPI (blue) in HEK293 biosensor cell lines transfected with constructs expressing 42, 20, 10, 5 Serine-Halo, or Halo alone. Enrichment of Halo signal in cytoplasmic tau aggregates is denoted with white arrows, enrichment in nuclear tau aggregates is denoted with orange arrows, and yellow arrows show lack of enrichment. (*B*) Quantification of the ratio of mean intensity of Halo within cytoplasmic tau aggregates relative to Halo signal in the surrounding cytoplasm for serine constructs in (*A*). n = 40 tau aggregates. Data represent mean and 95% CI. Statistics performed with one-way ANOVA. (**) *P* = 0.001; (****) *P* < 0.0001. (*C*) Immunofluorescence of tau-YFP (green), Halo (magenta), and DAPI (blue) in H4 neuroglioma cells expressing full length 0N4R P301S tau transfected with Halo and 42-serine-Halo. Cells were fixed and images 48 h post seeding.

We observed that 42 consecutive serine residues are sufficient to robustly target to both cytoplasmic and nuclear tau aggregates (Fig. 3*A*). We also observed significant enrichment of Halo in tau aggregates with 20-serine residues, and little to no enrichment with the 10 or 5-serine residues (Fig. 3*B*). Halo-tagged 42-serine was also robustly recruited to tau aggregates in H4 neuroglioma cells expressing full-length 0N4R P301S tau demonstrating this is not unique to HEK293 cells expressing the tau K18 fragment (Fig. 3*C*). Thus, polyserine alone is sufficient to mediate associations with tau aggregates in a length-dependent manner. This provides a molecular explanation for the recruitment of the polyserine-containing SRRM2 protein to tau aggregates in cell lines, mouse models, and patient samples (14, 17).

We also observed that 42, 20, 10, and 5-serine-Halo produced cytoplasmic foci that were not present in Halo alone (Fig. 3*A*), suggesting polyserine has self-assembly properties (see below).

### Cytoplasmic SRRM2 and PNN-Containing Assemblies Are Preferential Sites For Tau Aggregation.

To examine the temporal mechanisms through which SRRM2 co-localizes with cytoplasmic tau aggregates, we performed live imaging of HEK293 tau biosensor cells expressing endogenous SRRM2-Halo or PNN-Halo fusion proteins following seeding with tau transgenic mouse brain extracts. These experiments revealed the following key observations.

**Fig. 4.**
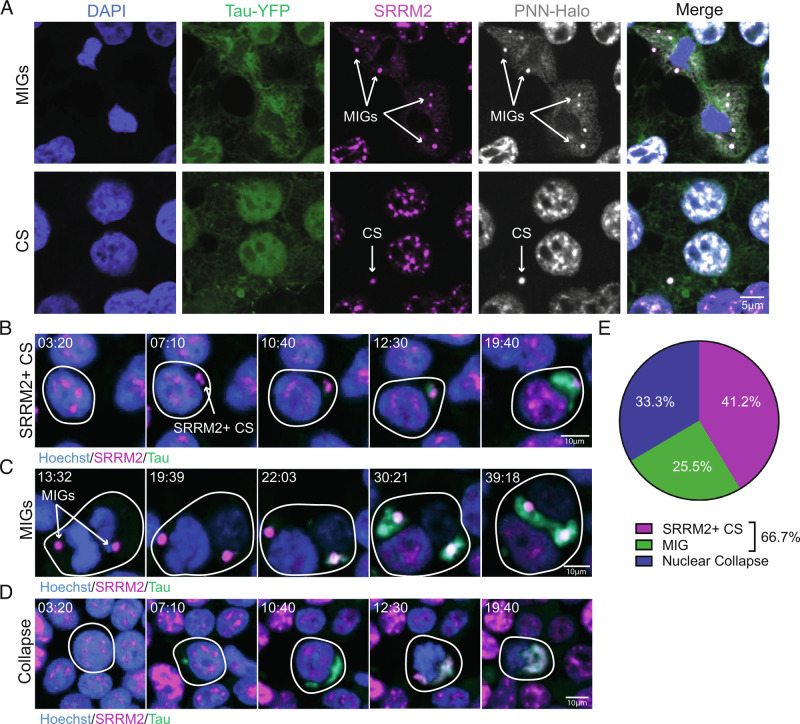
Endogenous SRRM2 assemblies associate with cytoplasmic tau aggregates. (*A*) Immunofluorescence of DAPI (blue), Tau-YFP (green), SRRM2 (magenta) and PNN-Halo (gray) showing the two types of SRRM2+ and PNN+ cytoplasmic assemblies: MIGs and CSs. MIGs are associated with cell division (defined by breakdown of nuclear membrane and chromatin condensation) and CSs are not associated with cell division (no evidence of nuclear membrane breakdown or chromatin condensation). (*B*–*D*) Live imaging of Hoechst (blue), Tau-YFP (green) and SRRM2_FL-Halo (magenta) in HEK293 tau biosensor cells seeded with tau aggregates and monitored for 48 h in 10-min increments. Stills from live imaging display tau aggregate formation at SRRM2 CSs (*B*) (Movie S3), MIGs (*C*) (Movie S4), and aggregate formation followed by nuclear collapse (*D*) (Movie S5). Time since the onset of imaging is displayed. (*E*) Quantification of the incidence of each mechanism from (*B*–*D*). 51 tau aggregates containing SRRM2 at the end of the movie were identified and then aggregates were scored by which mechanism led to the incorporation of SRRM2.

First, we observed that both SRRM2 and PNN can form two related transient cytoplasmic condensates. The first are MIGs—which contain nuclear speckle proteins—and form during mitosis following nuclear envelope breakdown (Fig. 4*A*) (25, 26). MIGs behave like typical condensates exhibiting a round shape and undergoing rapid fusion (Movie S1). We also observed SRRM2 and PNN stochastically form cytoplasmic assemblies independent of mitosis (Fig. 4*A* and Movies S2, S3, and S6). Similar cytoplasmic assemblies of SRRM2 have been previously observed in cultured cells and human neurons (27, 28). Since these assemblies contain SRRM2 and PNN which are typically found in nuclear speckles, we refer to these assemblies as cytoplasmic speckles (CSs).

Second, we observed two methods through which SRRM2 colocalized with cytosolic tau aggregates, which also illustrated how tau seeds propagate into larger aggregates in cells. In some cells, we observed the growth of tau aggregates that initiated at SRRM2+ CSs (Fig. 4*B* and Movie S3) or SRRM2+ MIGs (Fig. 4*C* and Movie S4). We quantified the percentage of tau aggregates that contained SRRM2 at the end of the video and found that 41.2% of aggregates began in close proximity (<2 µM) to CSs and 25.5% to MIGs. Collectively, these two mechanisms represent 66% of the SRRM2+ tau aggregates (Fig. 4*E*).

In the other cells, tau aggregates formed in the cytoplasm independently of any visible SRRM2+ condensate, followed by a rapid (<10 min) bulk movement of SRRM2 from the nucleus to the cytoplasmic tau aggregate, which we refer to as nuclear collapse (Fig. 4*D* and Movie S5, 33.3%). Whether these tau aggregates initiate on smaller SRRM2+ assemblies below the detection of light microscopy or in association with other subcellular structures remains to be assessed. Once detected, the rate of growth of tau aggregates (as assessed by total localized tau) was similar for tau aggregates that initiated in association with a MIG or CS as compared to tau aggregates that appeared to arise independently (*SI Appendix*, Fig. S3*D*). This suggests that association with a MIG or CS likely affects an early step in tau aggregate growth from transfected seeds.

We also observed similar results from live imaging of HEK293 tau biosensor cells with endogenously labeled PNN where either tau aggregation initiates at pre-existing PNN+ CSs or MIGs and remains associated while the tau aggregates are growing (*SI Appendix*, Fig. S3 *A* and *B* and Movies S6 and S7). PNN is also recruited to existing aggregates following nuclear collapse (*SI Appendix*, Fig. S3*C* and Movie S8).

These results identify MIGs and CSs as subcellular assemblies that are preferred sites for the propagation of tau aggregates, with this mechanism occurring in over half of all cases where tau aggregates contain SRRM2 (Fig. 4*E*). Strikingly, in cells that contain observable MIGs or CSs, we typically observe the initial growth of the tau aggregate occurring in conjunction with the MIG or CS. MIGs contain snRNAs (29), which is notable since tau aggregates in patients can contain U1 snRNAs (30, 31), and tau aggregates in cell lines and mouse models are enriched in snRNAs (14). This provides a possible explanation for why snRNAs and nuclear RBPs accumulate in tau aggregates in disease.

### Stress Granules Are Not Preferential Sites For Tau Seed Propagation into Aggregates.

Tau aggregates could preferentially form in association with MIGs and CSs due to their inclusion of RNA or due to other protein domains within these assemblies—such as polyserine domains—that may affect tau propagation. To test whether another cytoplasmic RNP assembly can also preferentially propagate tau seeds, we examined whether tau aggregates similarly formed in association with stress granules. Stress granules are assemblies of untranslating mRNPs that form when translation initiation is inhibited, and have been proposed to associate with pathological inclusions in neurodegenerative disease (32). We modified the HEK293 biosensor cells to stably express mRuby or mRuby-tagged G3BP1—a canonical stress granule marker. To induce stress granules that would persist during tau aggregation, we treated cells with Pateamine A (PatA), which inhibits translation initiation by disrupting eIF4A function (33).

Following the addition of tau seeds and 50 nM PatA, we observed stress granules and tau aggregates were mostly independent, with limited overlap or docking of mRuby-G3BP1 with tau aggregates only at a late timepoint (*SI Appendix*, Fig. S4 *A* and *B*). Live imaging of tau aggregate formation under conditions of PatA-mediated stress granule induction demonstrated tau aggregates formed independently of stress granules but could subsequently exhibit transient surface docking with stress granules (*SI Appendix*, Fig. S4*C* and Movies S9 and S10). Prior studies have shown tau associates in model systems and patient samples with TIA1, a nuclear protein that relocalizes to cytoplasmic stress granules under stress but—consistent with our findings—does not colocalize with other stress granule markers, most notably G3BP1 (34, 35). Collectively, these results highlight that the engagement of tau—and in the case of MIGs and CSs the preferential propagation of tau aggregates—with RNP granules is not a general property, but rather is specified by unique features of these assemblies.

### Cytoplasmic Assemblies Formed by Exogenous Polyserine-Containing Proteins Are Sufficient to Create Conducive Sites For Tau Aggregation.

**Fig. 5.**
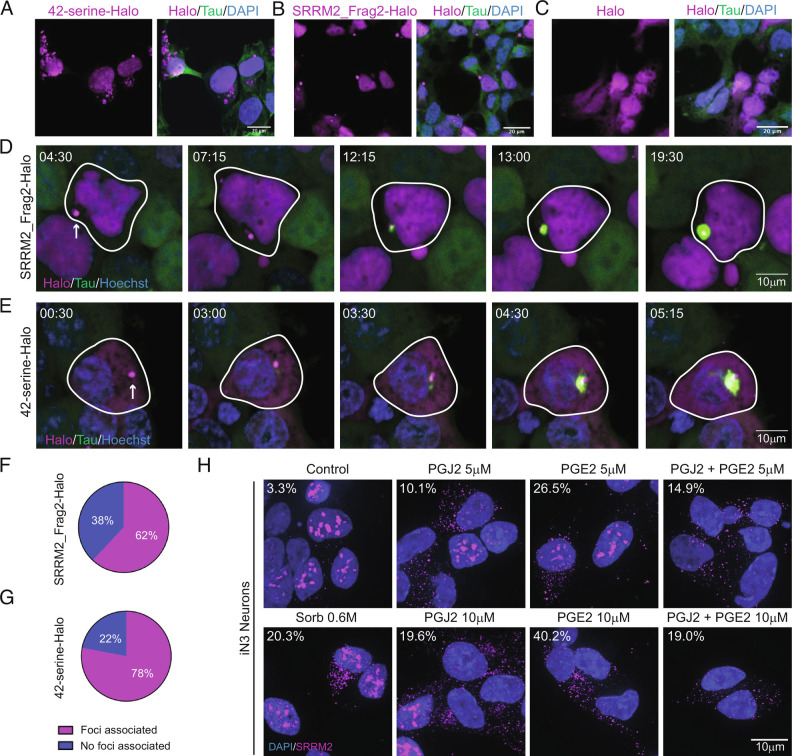
Polyserine-based assemblies are sites of tau aggregation. (*A*–*C*) Immunofluorescence of Halo (magenta), Tau-YFP (green) and DAPI (blue) in HEK293 biosensor cells transfected with 42-serine-Halo, SRRM2_Frag2-Halo, or Halo. (*D*) Stills from live-imaging of HEK293 tau biosensor cells expressing Tau-YFP (green), transfected with SRRM2_Frag2-Halo (magenta) and labeled with Hoechst (blue). Cells were lipofected with clarified tau brain homogenate and imaged for 24 h at a time interval of 15 min. Time since the onset of imaging is displayed. (Movie S11). (*E*) Stills from live-imaging of HEK293 tau biosensor cells expressing Tau-YFP (green), transfected with 42-serine-Halo (magenta) and labeled with Hoechst (blue). Cells were lipofected with clarified tau brain homogenate and imaged for 24 h at a time interval of 15 min. Time since the onset of imaging is displayed. (Movie S12). (*F* and *G*) Quantification of 50 tau aggregates that contained Halo signal at the termination of live imaging scored by whether tau aggregation initiated within 2 μM of cytoplasmic Halo+ foci in cells transfected with SRRM2_Frag2-Halo (*F*) or 42-serine-Halo (*G*). (*H*) Immunofluorescence of SRRM2 (magenta) with DAPI (blue) stain in human iPSC derived cortical neurons (iN3 neurons) at 12 d post-differentiation treated with either vehicle control, 0.6 M sorbitol for 1 h, PGJ2 for 15 h (5 μM or 10 μM), and/or PGE2 for 15 h (5 μM or 10 μM). Percentages represent the fraction of neurons with cytoplasmic SRRM2 foci post treatment.

Since polyserine domains are sufficient to interact with tau aggregates and are enriched in proteins found in nuclear speckles, MIGs, and CSs, we hypothesized that polyserine-containing protein domains, or polyserine itself, might be the driving principle for the formation of assemblies that serve as preferred sites for tau propagation. Consistent with this hypothesis, we observed that overexpression of 42-serine and SRRM2-Frag_2, but not Halo alone, led to the formation of cytoplasmic assemblies (Fig. 5 *A*–*C*).

Through live cell imaging, we observed tau aggregates preferentially formed in association with both SRRM2-Frag_2-Halo and 42-serine-Halo assemblies, similar to endogenously labeled assemblies of SRRM2 and PNN (Fig. 5 *D* and *E* and Movies S11 and S12). In transfected cells expressing SRRM2-Frag_2-Halo or 42-serine-Halo that had Halo+ assemblies, 62% and 78% of tau aggregates initiated in close proximity (<2 µM) to those Halo+ assemblies, respectively (Fig. 5 *F* and *G*). Thus, polyserine is sufficient to create assemblies that establish a local environment conducive to tau aggregation.

### Stress Induces CSs in iPSC Neurons.

The results above suggest that the formation of CSs in post-mitotic neurons might create condensates that would enhance the propagation of tau aggregates. Previous results have shown that amyloid-β toxicity can induce cytoplasmic assemblies of SRRM2, which we infer to be CSs, in mouse and human neurons (28). To determine if other stresses can induce CSs in post-mitotic neurons, we treated iPSC derived cortical neurons with various stressors (36, 37). We validated the neuronal identity of the iPSC-derived cortical neurons using two neuron-specific markers, β-III tubulin and MAPT (*SI Appendix*, Fig. S5 *A*–*C*) (36). We observed SRRM2+ CSs in neurons following hyperosmotic sorbitol stress, as well as inflammatory stress induced through treatment with prostaglandin J2 (PGJ2) and prostaglandin E2 (PGE2) (Fig. 5*H*), which are elevated in chronic inflammatory states and neurodegeneration (38–40). These results show external triggers, including aspects of neuroinflammation, can promote the formation of SRRM2+ CSs in neurons.

### Tau Aggregate Formation Is Modulated By Levels of Polyserine-Containing Proteins.

**Fig. 6.**
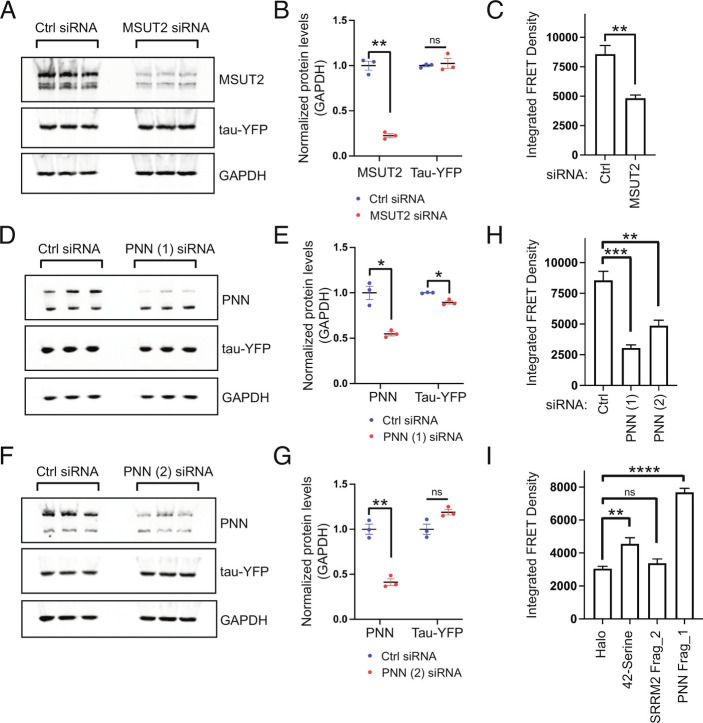
Cellular levels of polyserine-containing proteins modulate tau aggregation. (*A*) Western blot of MSUT2, tau-YFP and GAPDH protein levels in HEK293 biosensor cells treated with control or MSUT2 siRNA. siRNAs were transfected into HEK293 cells 48 h prior to transfection with tau seeds 24 h prior to analysis. (*B*) Quantification of Western blot shown in (*A*) normalized to GAPDH. (*C*) Integrated FRET density (product of FRET+ percentage and median fluorescence intensity) of HEK293 biosensor cells treated with control or MSUT2 siRNA measured by flow cytometry and analyzed as in *SI Appendix*, Fig. S6 *A*–*C*. (*D*) Western blot of PNN, tau-YFP and GAPDH protein levels in HEK293 biosensor cells treated with control or PNN (1) siRNA. (*E*) Quantification of Western blot shown in (*D*) normalized to GAPDH. (*F*) Western blot of PNN, tau-YFP and GAPDH protein levels in HEK293 biosensor cells treated with control or PNN (2) siRNA. (*G*) Quantification of Western blot shown in (*F*) normalized to GAPDH. (*H*) Integrated FRET density in HEK293 biosensor cells treated with control or each PNN siRNA measured by flow cytometry and analyzed as in (*A*–*F*). (*I*) Integrated FRET Density in HEK293 biosensor cells transfected with Halo, 42-Serine, SRRM2 Frag_2 and PNN Frag_1 constructs measured by flow cytometry and analyzed by single cell gating in *SI Appendix*, Fig. S6 *A* and *B*, subsequently Halo+ gating in *SI Appendix*, Fig. S7 *A* and *B*, and lastly for FRET positivity as in *SI Appendix*, Fig. S6*C*. Bars for all plots represent mean and SEM. Statistics for (*B*, *E*, and *G*) performed with unpaired *t* test with Welch’s correction. Statistics for (*C*) performed with Mann-Whitney test. Statistics for (*H*) and (*I*) performed with one-way ANOVA. (ns) *P* > 0.05; (*) *P* < 0.05; (**) *P* < 0.01; (****) *P* < 0.0001.

The results above suggest cytoplasmic assemblies enriched in polyserine domains form biochemical environments conducive to tau aggregation, which predicts that the level of polyserine repeat domains would correlate with tau aggregate formation. To examine this possibility, we increased or decreased the amount of polyserine regions in cells and examined tau aggregate formation in response to seeding. To quantify tau aggregation, we utilized FRET-based flow cytometry of the HEK293T tau biosensor cells as a measure of tau aggregation (24, 41). Cells were seeded with tau brain homogenate within a linear range (*SI Appendix*, Fig. S6*M*) and gating parameters were performed as previously reported leading to a shift of cells into the FRET+ population (*SI Appendix*, Fig. S6 *A*–*D*) (24). We validated this assay by showing the knockdown of MSUT2 (mammalian suppressor of tauopathy 2)—a nuclear speckle protein shown to alter tau aggregation in mouse and invertebrate model organisms (42)—led to a reduction in both the percentage of tau aggregate positive (FRET+) cells and the integrated FRET density (the product of FRET+ percentage and the median fluorescence intensity; FRET density is a combinatorial measure of the aggregation within each cell and a population-based analysis of the extent of tau aggregation) (42, 43) (Fig. 6 *A*–*C* and *SI Appendix*, Fig. S6*G*).

To reduce polyserine-containing domains in cells, we used siRNAs to knock down either SRRM2 or PNN. Successful knockdown of SRRM2—without a reduction in tau protein levels—showed no significant alteration in tau aggregation, as assessed by the percent of FRET-positive cells or integrated FRET density (*SI Appendix*, Fig. S6 *I*–*L*). However, since PNN (present at ~110,000 parts per billion) is more abundant than SRRM2 (present at ~60,000 parts per billion) (44), we hypothesized that SRRM2 knockdown may not sufficiently reduce levels of polyserine. To knock down PNN, we validated two siRNAs and monitored the effects on tau expression and tau aggregation (Fig. 6 *D*–*G*). While PNN (1) siRNA led to a modest reduction in tau levels, PNN (2) siRNA resulted in a slight increase (Fig. 6 *D*–*G*). Regardless of these effects on tau protein levels, knockdown with either siRNA led to a significant reduction in the percentage of FRET+ cells and integrated FRET density (Fig. 6*H* and *SI Appendix*, Fig. S6 *D*–*F* and *H*). Thus, a reduction in PNN levels reduces tau aggregation.

To determine whether increasing polyserine concentration affects tau aggregation, we transfected cells with constructs expressing Halo-tagged 42-polyserine, SRRM2-Frag_2, and PNN-Frag_1 and monitored tau aggregation via flow cytometry. An additional gating step to sort for transfected cells based on Halo expression was performed (*SI Appendix*, Fig. S7 *A* and *B*). We found overexpression of all three of these proteins resulted in an upward trend in FRET+ percentage with 42-serine and PNN-Frag_1 reaching statistical significance (*SI Appendix*, Fig. S7 *C*–*G*). Further, overexpression of 42-serine and PNN-Frag_1 also significantly increased integrated FRET density (Fig. 6*I*). Importantly, the expression of each construct did not lead to meaningful increases in tau levels as shown by the distribution of CFP signal in sorted cell populations (*SI Appendix*, Fig. S7 *H*–*K*).

In addition to PNN being more abundant than SRRM2, it is also possible that sequence or size differences between their serine-rich regions alter the accessibility and/or post-translational modifications of the regions leading to differential effects on tau aggregation when knocked down or overexpressed.

Taken together, these results indicate that decreasing or increasing polyserine domain levels in the cells can correspondingly decrease or increase tau aggregation. This provides evidence that polyserine-containing assemblies are not only sites of preferential tau aggregate growth, but their level can affect the degree of tau aggregation.

## Discussion

Our observations demonstrate that polyserine protein domains mediate interactions with tau aggregates. Polyserine-rich domains in the nuclear speckle proteins SRRM2 and PNN are necessary and sufficient for localization to tau aggregates (Figs. 1 and 2). Further, polyserine repeats alone are sufficient for targeting proteins to tau aggregates in a length-dependent manner (Fig. 3). The association of polyserine with tau aggregates provides a molecular explanation for the mislocalization of SRRM2 to pathogenic tau inclusions in postmortem samples from AD, CBD, and FTLD patients (14, 17).

**Fig. 7.**
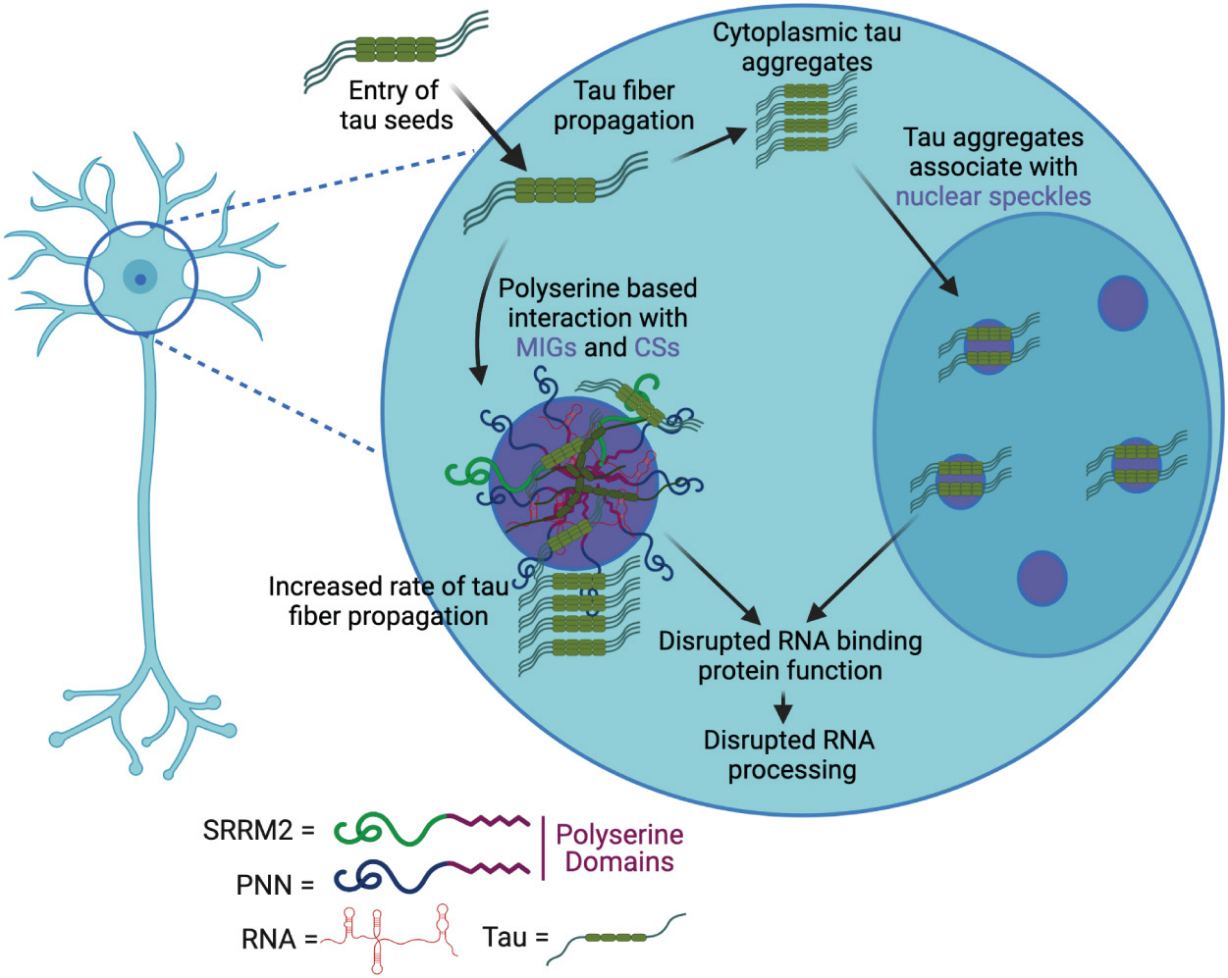
(*A*) Model showing a possible mechanism by which tau seeds introduced into HEK293 tau biosensor cells can form cytoplasmic tau aggregates or be recruited to SRRM2+ and PNN+ CSs or MIGs that contain polyserine domains and can be derived from nuclear speckles. Tau aggregates can grow and propagate within MIGs and CS. Tau’s interaction with RBPs such as SRRM2 and PNN in MIGs, CSs, and nuclear speckles could induce a loss of function in RBP function and lead to disruptions in RNA processing.

Additional evidence demonstrates that polyserine domains are sufficient to create biological assemblies that are preferential sites of tau aggregate propagation. First, we observed that in a seeding model, 66% of tau aggregates that are SRRM2+ initiate in association with either MIGs or CSs (Fig. 4). Second, overexpression of either the SRRM2 C-terminal domain containing polyserine runs (SRRM2-Frag_2-Halo), or 5, 10, 20, or 42-polyserine, drives the formation of assemblies in cells and similar results can be seen in vitro for 42-polyserine, demonstrating polyserine has self-assembly properties (Fig. 5). Third, the condensates formed by the overexpression of 42-polyserine can also serve as preferred sites of tau aggregation (Fig. 5). Fourth, the knockdown of PNN leads to a reduction in the formation of tau aggregates (Fig. 6). Finally, we observed overexpression of 42-polyserine or the PNN C-terminus (PNN-Frag_1-Halo) increased tau aggregation (Fig. 6). A unifying model is that polyserine domains can self-assemble and define a biochemical environment that can promote tau aggregate growth (Fig. 7).

The observation that MIGs, CSs, or artificial polyserine-based cytoplasmic assemblies serve as preferred sites of tau aggregation provides an explanation for nuclear tau aggregates forming in nuclear speckles, which are another subcellular domain rich in polyserine-containing proteins (*SI Appendix*, Table S1) (14). Moreover, a 42-serine repeat is sufficient to target exogenous proteins to nuclear tau aggregates (Fig. 3*A*, orange arrows), demonstrating the molecular interactions between polyserine and tau are similar in both cytoplasmic and nuclear tau aggregates.

This work illustrates the fundamental principle that the growth of aberrant protein aggregates can be enhanced by specific subcellular locations with defined biochemical composition. Our results indicate the effects of polyserine-containing assemblies are not a general principle of RNP assemblies as G3BP1+ stress granules do not associate with tau aggregates during formation (*SI Appendix*, Fig. S4). In principle, a polyserine-defined biochemical environment could enhance the rate of tau aggregate growth in several, possibly overlapping, manners. By interacting with tau monomers, it could increase the local concentration of tau and the probability of tau-tau interactions. One attractive possibility is that polyserine is sufficient to nucleate a condensate that recruits tau and additional factors to promote aggregation as has been seen with other aggregation-prone proteins (45–47). Alternatively, polyserine interaction with tau could stabilize a particular fold of either the tau monomer or seed that enhances aggregate growth. For example, since polyserine can form a type of aggregate related to a coiled-coil (48), it could provide a template for the increased rate of tau aggregate growth. Another possibility is that polyserine, or a polyserine-interacting molecule, could serve as a co-factor for tau propagation. Interestingly, since polyserine domains can be heavily phosphorylated (49), a phosphorylated polyserine region could function similarly to other polyanions such as RNA or heparin that promote tau fibrillization in vitro (10–12). Further work will be necessary to elucidate the mechanisms through which polyserine regulates tau aggregation.

Multiple observations suggest that the interaction between polyserine domains and tau will be pertinent to human disease. First, SRRM2 is known to mislocalize to tau aggregates in postmortem samples from AD, CBD, and FTLD patients (14), and the degree of mislocalization corresponds with increased severity of pathological tau deposition in humans and mouse models (17). Second, previous work has shown that β-amyloid deposition can promote SRRM2 phosphorylation and export to the cytoplasm leading to disruptions in neuronal RNA splicing (28). Third, we observed SRRM2+ CSs form in non-dividing human iPSC-derived cortical neurons when stressed with the inflammatory compounds PGJ2 and PGE2 as well as sorbitol-induced hyperosmolar stress (Fig. 5). This suggests that β-amyloid abnormalities, or other inflammatory triggers, could promote the movement of SRRM2 and PNN to the cytoplasm, promote CS formation, and lead to increased tau aggregate growth. This could provide a molecular link explaining how β-amyloid plaques and other inflammatory compounds can increase the probability of tau tangle formation in human tauopathies.

The polyserine-mediated interactions of tau with nuclear speckle components, either in the nucleus or the cytosol may contribute to neurotoxicity by disrupting RNA homeostasis. One possible mechanism of neurotoxicity is the sequestration of SRRM2 or PNN in cytoplasmic tau aggregates leading to the loss of their physiologic function. SRRM2 is highly conserved throughout evolution and loss of function mutations in SRRM2 have been shown to cause neurodevelopmental disorders suggesting that perturbations leading to SRRM2 loss of function are deleterious to neurons (50). Additional evidence suggests that loss of SRRM2’s nuclear function leads to splicing abnormalities through proteins such as PQBP1 (28). Interestingly nuclear speckles persist despite loss of SRRM2, and this is most likely due to SON’s role in the nuclear speckles (22) and SON’s lack of interactions with tau aggregates (14). Furthermore, deletion of the nuclear speckle protein MSUT2 (14, 43) has been shown to suppress tau toxicity in mouse models and decreases tau aggregates in cells (42) (Fig. 6) further connecting tau toxicity to nuclear speckle components.

Taken together, we suggest a working model for the relationship between tau aggregation and RNP granules with the following key principles (Fig. 7). First, when tau seeds form or enter a cell, they can be cleared, or initiate new aggregates with or without an association with MIGs and CSs. We hypothesize that the probability of tau aggregate growth will be increased in neurons with SRRM2+ and/or PNN+ CSs. Conditions that increase CSs, such as the presence of high concentrations of neuroinflammatory compounds like PGE2 and PGJ2 (Fig. 5), or proximity to amyloid-β plaques (28) could increase tau propagation in neurons. An important continuation of this study will be to determine what additional extracellular or intracellular events increase CS formation in post-mitotic neurons and assess how those events influence tau propagation and downstream mechanisms of neurodegeneration.

## Materials and Methods

Cell lines were maintained at 5% CO_2_ at 37 °C in Dulbecco’s modified Eagle’s medium supplemented with 10% fetal bovine serum and 1% penicillin/streptomycin. Stable cell lines were generated using lentiviral transduction. IF was performed as described by Lester et al. (14). A complete description of the *Materials and Methods* can be found in the *SI Appendix*, *SI Materials and Methods*.

## Supplementary Material

Appendix 01 (PDF)

Movie S1. Movie showing tau aggregate (tau-YFP, green) nucleation from SRRM2+ MIGs (SRRM2-Halo, red).

Movie S2. Movie showing a stable SRRM2+ CS (SRRM2-Halo, red) in the cytoplasm of a HEK293 cell independent of cell division.

Movie S3. Movie showing tau aggregate (tau-YFP, green) nucleation from an SRRM2+ CS (SRRM2-Halo, red).

Movie S4. Movie showing tau aggregate (tau-YFP, green) nucleation from SRRM2+ MIGs (SRRM2-Halo, red).

Movie S5. Movie showing cell collapse where SRRM2-Halo (red) quickly merges with a tau aggregate (tau-YFP, green).

Movie S6. Movie showing tau aggregate (tau-YFP, green) nucleation from PNN+ CS (PNN-Halo, red).

Movie S7. Movie showing tau aggregate (tau-YFP, green) nucleation from PNN+ MIG (PNN-Halo, red).

Movie S8. Movie showing cell collapse where PNN-Halo (red) quickly merges with a tau aggregate (tau-YFP, green).

Movie S9. Movie showing tau aggregates (tau-YFP, green) forming independently of G3BP1-Halo (red)

Movie S10. Movie showing tau aggregates (tau-YFP, green) forming independently of G3BP1-Halo (red). Example 2

Movie S11. Movie showing tau aggregates (tau-YFP, green) nucleating from SRRM2ct-Halo (red) MIGs.

Movie S12. Movie showing tau aggregates (tau-YFP, green) nucleating from 42serine-Halo (red) CSs.

## Data Availability

All study data are included in the article and/or SI Appendix.
