# Supplementary material for: Cytosolic condensates rich in polyserine define subcellular sites of tau aggregation
Source: Proc Natl Acad Sci U S A. 2023 Jan 10;120(3):e2217759120. doi: 10.1073/pnas.2217759120 (PMC9934293; doi:10.1073/pnas.2217759120)
Supplement: Supplementary file 1 — Appendix 01 (PDF) [file pnas.2217759120.sapp.pdf]

PNAS

Supporting Information for

Cytosolic condensates rich in polyserine define subcellular sites of tau aggregation

Evan Lester[a,b,1], Meaghan Van Alstyne[b,c,1], Kathleen L. McCann [b,c], Spoorthy Reddy[b], Li Yi Cheng[b], Jeff Kuo[b], James Pratt[b], Roy Parker[b,c,*]
[a]Medical Scientist Training Program, University of Colorado Anschutz Medical Campus, Aurora, CO, USA
[b]Department of Biochemistry, University of Colorado Boulder, CO, USA
[c]Howard Hughes Medical Institute, University of Colorado, Boulder, CO, USA
1: Contributed equally
*Corresponding author: Roy Parker
Email: Roy.Parker@Colorado.edu

This PDF file includes:

Materials and Methods
Figures S1 to S7
Legend for Table S1
Legends for Movies S1 to S12

Other supporting materials for this manuscript include the following:

Table S1
Movies S1 to S12

MATERIALS AND METHODS

Cell culture and tau aggregate seeding of HEK293 cells

As previously described (4, 14), HEK293 biosensor cells stably expressing the 4R repeat domain of tau (K18) with the P301S mutation were purchased from ATCC (CRL-3275) (previously described in (23)). Cells were seeded at 1.25 x 10^5 cells/mL in 500uL of DMEM with 10% FBS and 0.2% penicillin-streptomycin antibiotics on PDL-coated glass coverslips in a 24-well tissue culture treated plate (Corning 3526) and allowed to grow overnight in incubators set to 37°C with 5% carbon dioxide. The next day, 7ug of 1 mg/mL clarified P301S tau mouse brain homogenate or 7uL PBS was mixed with 6uL of Lipofectamine 2000 and brought up to 100uL in PBS and allowed to sit at room temperature for 1.5 hours. The mixture was then added to 300uL of DMEM without FBS or antibiotics and mixed by pipetting. 50uL of this mixture was added to each well of a 24-well plate and allowed to incubate at 37 °C for 24 hours. Tau aggregate formation was monitored using a fluorescence microscope with a 488nm filter.

Cell culture and tau seeding of H4 biosensor cells

As previously described (14), H4 cells (ATCC Cat# HTB-148, RRID:CVCL_1239) stably expressing the pIRESpuro3 vector (Clontech) containing a codon-optimized 0N4R MAPT gene with the P301S point mutation and tagged with YFP were cultured in Dulbecco's Modified Eagle's Medium (DMEM) supplemented with 10% fetal bovine serum (FBS) and 0.2% penicillin-streptomycin, and maintained in incubators set to 37°C with 5% carbon dioxide. Cells were plated in a 12-well glass-bottomed dish at 1.25x10^5 cells/well and allowed to settle for a minimum of 2 hours prior to infection with tau seeds (described above). Cells were grown for 48 hours in the presence of tau seeds prior to fixation.

Clarification of brain homogenate for tau aggregate seeding in HEK293 cells

As previously described (14), 10% brain homogenate from Tg2541 or WT mice was centrifuged at 500 x g for 5 minutes, and the supernatant was transferred to a new tube and centrifuged again at 1,000 x g for 5 minutes. The supernatant was again transferred to a new tube and the protein concentration was measured using bicinchoninic acid assay (BCA) and diluted in DPBS to 1 mg/mL for transfection into HEK293 tau biosensor cells.

Immunofluorescence

As described above, cells were grown in 24 well plates on PDL-coated coverslips, fixed for 10 minutes in 4% paraformaldehyde, washed 3x with PBS, and permeabilized with 0.1% Triton-X100 for 10 minutes. Cells were then washed with PBS 3X, blocked in 5% BSA for 30 minutes, followed by the addition of primary antibodies (Supplemental Table 2) at indicated concentration in 5% BSA, and incubated overnight at 4 deg on a rotator. Cells were washed 3X with PBS and incubated with secondary antibody in 5% BSA for 30 minutes at room temperature

on a rotator. Cells were then washed 3X with PBS and incubated in DAPI for 5 minutes at a final concentration of 1μg/mL in PBS. Cells were washed one more time with PBS and mounted using ProLong Glass Antifade Mountant.

Generation of cell lines

To generate CRISPaint edited cell lines, HEK293 tau biosensor cells were seeded at $5*10^5$ cells/ml in 6 well plates and allowed to grow overnight in a 37°C incubator. The next day, Lipofectamine3000 was used to transfect cells with 0.5ug of pSpCas9(BB)-2A-GFP(PX458) targeting plasmid (Addgene # 48138) containing sgRNA sequences targeting full-length or truncations in SRRM2 and PNN (Supplemental Table 3), 0.5ug of the respective pCAS9-mCherry-Frame selector plasmid (Addgene #66939, 66940, 66941), and 1μg of pCRISPaint-HaloTag-PuroR plasmid (Addgene #80960). After 24 hours, cells were selected with 2ug/mL puromycin. JF646 was added to the media at a final concentration of 200nM for 24 hours to covalently label the Halo fusion proteins for visualization by fluorescence imaging and analysis by gel electrophoresis.

For generation of mRuby-tagged cell lines, constructs encoding mRuby2 or mRuby2-G3BP1 in a pLenti EF1 vector backbone were cloned. HEK293T WT cells were transfected using Lipofectamine3000 with pLenti plasmids and lentiviral packaging plasmids (Gag-pol (Addgene #14887), VSV-G (Addgene #8454), rSV-Rev (Addgene #12253)). Lentivirus was used to transduce HEK293 tau biosensor cells followed by selection with blasticidin (2μg/mL).

Cloning and expression of SRRM2 and PNN C-terminal fragments and polyserine repeats

To express SRRM2 C-terminal fragments in a pcDNA-PuroR expression plasmid, RNA was extracted from SRRM2_FL-Halo cells using Trizol, and reverse transcribed to cDNA using oligodT primers and Superscript III. Regions in the C-terminus of SRRM2_FL-Halo were amplified by PCR and cloned into EcoRV/XbaI digested pcDNA plasmid using In-Fusion cloning. For polyserine-Halo and PNN_Frag1 constructs, gene blocks were ordered from IDT with codons optimized for synthesis then cloned via In-Fusion into the pcDNA plasmid backbone.

Gel electrophoresis and Western blotting

For analysis of SRRM2 truncation cell lines, cells were grown in a 6-well plate to 50-80% confluence, washed 1x with PBS, and trypsinized in 0.5mL of trypsin. Cells were collected in a 1.5mL microcentrifuge tube and centrifuged at 500g for 5 minutes, washed 1x with PBS, and brought up in 100uL of lysis buffer (25mM Tris pH 7.5, 5% glycerol, 150mM NaCl, 2.5mM $MgCl_2$, 1% NP-40, 1:20 BME, 1X phosphatase/protease inhibitor). The lysate was pipetted up and down to mix and incubated on ice for 5 minutes. The lysate was then centrifuged at 16,000g for 5 minutes and the supernatant was transferred to a new tube and protein concentration was measured via Bradford. 10-15 ug of protein was combined with 4X LDS loading dye and boiled for 7 minutes prior to loading on a NuPAGE 4 to 12% Bis-Tris mini protein gel. Gels were directly imaged to examine covalently linked JF646.

For validation of PNN CRISPaint edited cell lines, $6x10^5$ cells were plated in a 6-well format 24 hours before addition of JF646 was added at a final concentration of 200uM and collected 24 hours after labeling. Cell pellets were lysed in 2X SDS loading buffer, passed through a 25G syringe, and boiled. Extracts were run on 4-20% Tris-Glycine protein gel and imaged directly for JF646 fluorescence. The gel was then transferred using iBlot 2 Transfer Device (Thermo Fisher) to nitrocellulose membranes for Western blotting.

For Western blotting, membranes were blocked in 5% milk in Tris-buffered Saline with 0.1% Tween (TBS-T) for 1 hour, incubated with primary antibodies (Supplemental Table 2) in TBS-T for 2 hours at room temperature, washed 3 x 10 minutes with TBS-T, incubated with secondary antibodies in TBS-T for 1 hour at room temperature, then washed 6 x 5 minutes with TBS-T before developing with Clarity Western ECL Substrate (Bio-rad).

Live cell imaging

For analysis of tau aggregate formation and SRRM2 relocalization, HEK293 tau biosensor cells with SRRM2_FL-Halo were seeded in 24 well glass bottom plates with #1.5 cover glass at $2.5*10^5$ cells/ml with or without 200nM JF646-Halo ligand and allowed to grow overnight at 37°C. To counter stain nuclei, Hoechst 33342 was added to the cell culture media and allowed to incubate for 15 minutes prior to imaging. For analysis of SRRM2 relocalization during tau aggregation, cells were imaged on an Opera Phenix High Content imaging system where images in the Cy5 (Halo-JF646), GFP (Tau-YFP), and DAPI (Hoechst) channels were acquired every 10 minutes for 48 hours at 37°C and 5% CO_2.

For live imaging of tau aggregate formation and PNN relocalization, HEK293 tau biosensor cells with PNN_FL-Halo labelling were seeded at a density 1.25×10^5 cells per 24-well in poly-L-lysine coated glass bottom plates. The following day, media was changed to Fluorobrite DMEM supplemented with 10% FBS and seeded using Lipofectamine3000 with tau brain homogenate. 5 hours post-seeding JF646 ligand (200nM) and Hoechst 33342 were added. 1-hour post-labeling imaging was started using a Nikon Spinning Disk Confocal acquiring images every 10 minutes for 24 hours.

For live imaging of tau aggregate formation and stress granules, HEK293 tau biosensor cells with mRuby2-G3BP1 were plated and seeded as described for PNN-Halo live imaging. 5 hours post-seeding Hoechst 33342 (1μg/mL) and Pateamine A (50nM) were added and after 30 minutes imaging was initiated with acquisition every 10 minutes for the following 24 hours.

For live imaging of transiently transfected 42-serine and SRRM2_Frag2-Halo constructs and tau aggregate formation, HEK293 tau biosensor cells were seeded at a density of 1.00×10^5 cells per 24-well in poly-L-lysine coated glass bottom plates. The following day, cells were transfected using Lipofectamine3000 with 500ng plasmid per well. 24 hours post-transfection cells were seeded with tau brain homogenate. 5 hours post-seeding JF646 ligand (200nM) and Hoechst 33342 were added. 1-hour post-labeling imaging was started using a Nikon Spinning Disk Confocal acquiring images every 15 minutes for 24 hours.

Protein expression and purification

A gene block for codon optimized 42-serine-Halo and Halo were purchased from IDT and used to subclone 42-serine-Halo and Halo into pET28a plasmid modified to have an N-terminal 6xHis-SUMO tag. SUMO-Halo and SUMO-42-serine-Halo were expressed in Rosetta2(DE3)pLysS e. coli in LB for 4 hours at 37°C after induction with 200 uM IPTG. The cell pellets were resuspended in lysis buffer (50 mM MOPS pH 7.0, 300 mM NaCl, 0.1% NP-40, 30 mM Imidazole, 1 mM DTT) supplemented with complete ULTRA EDTA-free protease inhibitors and 0.1 mM AEBSF and lysed by sonication. The lysate was labeled with JF549 Halo ligand for 30 min at room temperature. Proteins were purified using Ni2+-NTA resin and eluted with 50 mM MOPS pH 7.0, 300 mM NaCl, 0.1% NP-40, 300 mM Imidazole, and 1 mM DTT supplemented with complete ULTRA EDTA-free protease inhibitors and 0.1 mM AEBSF. Eluted proteins were dialyzed overnight at room temperature into 50 mM MOPS pH 7.0, 100 mM NaCl, 0.1% NP-40, and 1 mM DTT.

***In vitro* polyserine assembly**

To assess assembly formation, 20 µM purified SUMO-Halo and SUMO-42-serine-Halo were incubated with TEV for 22 hours at room temperature in a dialysis buffer. Reactions were either subjected to ultracentrifugation, microscopic examination or boiled in SDS and analyzed by SDS-PAGE. For ultracentrifugation analysis, reactions were spun at 100,000g for 1 hr, washed with dialysis buffer, and spun a second time at 100,000g for 30 min. 1 µl samples from the total, supernatant and pellet fractions were spotted onto nitrocellulose and blotted with rabbit anti-HaloTag pAB (Promega). Reactions were transferred to Bio-One CELLview slides (Greiner) and imaged at 60X magnification on Nikon epifluorescence microscope. For SDS-PAGE analysis, reactions were boiled in 1x SDS-loading dye for 5 min at 95°C, run on a 4-12% Bis-Tris gel, and transferred to nitrocellulose. The membrane was blotted with rabbit anti-HaloTag pAB (Promega).

Differentiation and Stress of iPSC-derived Cortical Neurons

As previously described (35), WTC-11 cells expressing a doxycycline-inducible form of the master neuronal transcriptional regulator neurogenin-2 (NGN2) (these cells are also known as i^3Neurons) were thawed onto vitronectin-coated 6 well tissue culture plates in E8 culture medium supplemented with 10 uM ROCK inhibitor. The next day, the E8 medium with ROCK inhibitor was removed and replaced with E8 medium without ROCK inhibitor. The cells were expanded and passaged using 500uM EDTA until colonies reached a confluency of 70-80% in 10cm tissue culture plates. Once at 70-80% confluence, iPSCs were split using Accutase to get a single cell suspension and 2-2.5x10^7 cells were transferred onto Matrigel coated 15cm tissue culture dishes containing Neuronal Induction Media with doxycycline (see Supplementary table 4 for details). Cells were grown at 37°C and Neuronal Induction Media with doxycycline was changed daily for 3 days. After 3 days of induction, neurites were clearly visible, and the neurons were split using accutase and replated onto PDL-coated XonaChip Imaging Slides (Fisher

Scientific NC1648769) at a density of 1-5x10^5 cells/mL in Neuronal Culture Media (see Supplementary Table 4 for details). Cells were then allowed to grow for 7 days prior to experimentation with Neuronal Culture Media changed every 3 days. During this period, cells were checked daily under a phase-contrast microscope to ensure neuronal health.

To stress cells, media was removed and replaced by Neuronal Culture Media containing the appropriate concentration of prostaglandin J2, E2, J2 + E2 (5uM or 10uM). After 15 hours, the cells were fixed with 4% PFA and IF performed as described above using the anti-SRRM2 antibody and DAPI. For the 0.6M Sorbitol condition, cells were treated for 1 hour rather than 15 hours.

siRNA transfections

For siRNA validation, HEK293 tau biosensor cells were plated at a density of 7.0x10^5 cells per 6-well. The following day cells were transfected with 25 pmol siRNA with Lipofectamine RNAiMAX and collected 60 hours post-transfection. Cell pellets were lysed in 2X SDS loading buffer and processed for Western blotting as described above.

For flow cytometry, HEK293 tau biosensor cells were transfected with siRNA as described. 24 hours post-transfection cells were plated at a density of 1.25x10^5 cells/ 24-well. 24 hours later cells were seeded with tau brain homogenate for 24 hours prior to analysis. siRNAs used are detailed in Supplemental table 5.

Flow cytometry

For siRNA experiments, HEK293T tau biosensor cells were transfected with siRNAs 48 hours prior to seeding with tau brain homogenate at a final concentration of 0.5ng/μl. For overexpression experiments, HEK293T tau biosensor cells were transfected with plasmids 24 hours prior to seeding with tau brain homogenate (0.5 ng/μl) and addition of TMRDirect Halo ligand (200nM) to label exogenously expressed Halo-tagged fusion proteins. 24 hours post-seeding, cells were trypsinized, washed with PBS, and filtered with 50um nylon mesh filters prior to cell sorting. Sorting was performed with a BD FACSCelesta^{TM} Cell Analyzer using the following filter sets: 561-585 (Halo), 405-450 (CFP), and 405-525 (FRET). Analysis was performed using FlowJo. Gating was performed in sequential steps, first sorting for cells, single cells, then (when applicable) gating based on Halo expression was performed. Lastly, gating for FRET+ cells was performed based on mock seeded cells to set a false FRET percentage at 1 as previously detailed (40). Integrated FRET Density was calculated as a product of the percentage of FRET-positive cells and median fluorescence intensity.

Image analysis using Ilastik and CellProfiler

To measure the enrichment of endogenous SRRM2, C-terminal fragments, polyserine, and controls in tau aggregates, images were first segmented into cytoplasmic tau aggregates, nuclear tau aggregates, nucleus, cytosol, and background using Ilastik with a minimum of five training images per condition per experiment. Images were hand-annotated to show the location of the desired

structures, which enabled the construction of a model that could then segment subsequent images. The original image and the image segmentation masks created by Ilastik were then used as inputs for a CellProfiler pipeline that calculated pixel intensity values of the 488 and 647 channels within the masked compartments. These compartmental measurements were used to calculate the enrichment of SRRM2 in tau aggregates per image as follows: cytoplasmic tau aggregate enrichment = mean intensity within cytoplasmic tau aggregates per image / mean intensity within the cytosol per image.

To measure the enrichment of endogenous PNN or its polyserine-rich sequence and controls in tau aggregates, a CellProfiler pipeline was generated to segment the cytoplasm and nuclei of individual cells. Mean intensity measurements were taken and the fold enrichment reported for each cell as the mean intensity of Halo signal within tau aggregates / mean intensity of the remainder of the cytoplasm. This method was also used to quantify the fold enrichment of mRuby2 or mRuby2-G3BP1 signal in tau aggregates.

Protein name(s)	Uniprot ID	subcellular location	Expressed in neurons?	Expressed in HEK293?	Serine locus size	# of serines in locus	% serine in locus	Longest consecutive serine run	Localization to tau aggregates in HEK293
TNRC18; CAGL79	O15417	Nucleoplasm	Yes (>20 nTPM)	Lowly (<20 nTPM)	61	59	97%	58	Untested
CHD9	Q3L8U1	Nucleoplasm, cytosol	Yes (>20 nTPM)	Lowly (<20 nTPM)	66	51	77%	11	Untested
PNN; DRSP; MEMA	Q9H307	Nuclear speckles	Yes (>20 nTPM)	Yes (>20 nTPM)	53	44	83%	11	Yes
MLLT3; Protein AF-9	P42568	Nucleoplasm, cytosol	Yes (>20 nTPM)	Yes (>20 nTPM)	46	44	96%	42	Untested
SRRM2; SRM300; SRL300; KIAA0324	Q9UQ35	Nuclear speckles	Yes (>20 nTPM)	Yes (>20 nTPM)	44	43	98%	42	Yes
RLIM; RNF12	Q9NVW2	Nucleoplasm, cytosol	Lowly (<20 nTPM)	Yes (>20 nTPM)	38	31	82%	20	Untested
SETD1A; SET1; SET1A; KMT2F; KIAA0339	O15047	Nuclear speckles	Lowly (<20 nTPM)	Yes (>20 nTPM)	34	30	88%	24	Yes
HCG1998636; isoform CRA_a	A0A024QZB1	Unknown	unknown	unknown	34	29	85%	24	Untested
ZBTB4	Q9P1Z0	Nucleoplasm, cytosol	Yes (>20 nTPM)	Lowly (<20 nTPM)	27	26	96%	16	Untested
ZNF865	P0CJ78	Nucleoli, Nucleoplasm	Lowly (<20 nTPM)	Lowly (<20 nTPM)	25	25	100%	25	Untested
DACH1	Q9UI36	Nuclear speckles, nucleoplasm	Yes (>20 nTPM)	Lowly (<20 nTPM)	27	25	93%	24	Untested
ARL6IP4; AIP-4; SRrp37; SR-25; HSP-975	Q66PJ3	Nucleoplasm, mitochondria	Yes (>20 nTPM)	Yes (>20 nTPM)	25	25	100%	25	Untested
MBTPS2	O43462	Nucleoplasm, cytosol, mitochondria	Yes (>20 nTPM)	Lowly (<20 nTPM)	26	25	96%	23	No
PPRC1; PRC; KIAA0595	Q5VV67	Nucleoplasm	Lowly (<20 nTPM)	Yes (>20 nTPM)	29	24	83%	20	Untested
KCNMA1; Slo1, MaxiK; hSlo; SAKCA	Q12791	Untested	Yes (>20 nTPM)	No (<1 nTPM)	26	23	88%	22	Untested
TMEM40	Q8WWA1	Cytosol	No (<1 nTPM)	No (<1 nTPM)	23	22	96%	21	Untested
PRDM2; KMT8; RIZ	Q13029	Nucleoplasm, Golgi apparatus	Yes (>20 nTPM)	Lowly (<20 nTPM)	24	22	92%	13	Untested
NAF1	Q96HR8	Nucleoplasm, cytosol	Yes (>20 nTPM)	Yes (>20 nTPM)	20	19	95%	18	Untested

Table S1.
Table shows all human proteins containing serine regions >20 amino acids with more than 75% serine composition.

Table S2. Antibodies

Antibody name	Manufacturer	Catalog #
Rabbit anti-SRRM2	ThermoFisher	PA5-66827
Rabbit anti-PNN (Pinin)	ThermoFisher	18266-1-AP
Rabbit anti-SETD1A	Millipore Sigma	HPA058376
Rabbit anti-Halo	Promega	G9281
Mouse anti-Tau-5	Thermo Fisher	AHB0042
Rabbit anti-B-III Tubulin	Cell Signaling	5568T
Rabbit anti-MSUT2	Sigma-Aldrich	HPA049798
GAPDH-HRP	Santa Cruz	sc-47724 HRP

Table S3. CRISPaint sgRNA Sequences

Name	Sequence (5'-3')	Frame selector
SRRM2_FL_for	CACCGCCATGAGACACCGCTCCTCC	0
SRRM2_FL_rev	AAACGGAGGAGCGGTGTCTCATGGC	
SRRM2_1_for	CACCGCTAGAGCGCCTCTTCCGTT	2
SRRM2_1_rev	AAACAACGGAAGAGGCGCTCTAGC	
SRRM2_2_for	CACCGATTGGTCTGAAAAAGCAGA	0
SRRM2_2_rev	AAACTCTGCTTTTTCAGACCAATC	
SRRM2_3_for	CACCGCTTGCTGCAGGACAGACAT	0
SRRM2_3_rev	AAACATGTCTGTCCTGCAGCAAGC	
SRRM2_4_for	CACCGTTTCCAGCTGATTAAGAAA	0
SRRM2_4_rev	AAACTTTCTTAATCAGCTGGAAAC	
SRRM2_5_for	CACCGGCGTTCTAGATTTAGCTTT	0
SRRM2_5_rev	AAACAAAGCTAAATCTAGAACGCC	
SRRM2_6_for	CACCGGTCTAGGTCAGCAAGGCGA	0
SRRM2_6_rev	AAACTCGCCTTGCTGACCTAGACC	
SRRM2_7_for	CACCGCTGGCATGCCGAGAAACTT	2
SRRM2_7_rev	AAACAAGTTTCTCGGCATGCCAGC	
PNN_FL_for	CACCGAAAATCAGACAGGAAAGACAAA	0
PNN_FL_rev	AAACTTTGTCTTTCCTGTCTGATTTTC	
PNN_ΔCt_for	CACCGAAAACTAGGAGCAGAAGTAG	1
PNN_ΔCt_rev	AAACCTACTTCTGCTCCTAGTTTTC	

Table S4. iPSC/Neuronal culture reagents

Reagent	Manufacturer	Catalog #	Stock [ ]	Final [ ]
Essential 8 Media	Thermo Fisher	A1517001	N/A	N/A
ROCK inhibitor	Tocris	1254	10mM	10uM
Vitronectin	Thermo Fisher	A14700	100X	1X
Matrigel	Corning	354277	Per spec sheet	Per spec sheet
EDTA, pH 8.0	Invitrogen	AM9260G	500mM	500uM
Accutase	Fisher Scientific	NC9971356	N/A	N/A
**Neuronal Induction Media**				
DMEM/F12, HEPES	Gibco	11330032	N/A	N/A
N2 Supplement	Gibco	17502048	100X	1X
Non-essential amino acids	Gibco	11140050	100X	1X
L-glutamine	Gibco	25030081	100X	1X
Doxycycline	Sigma	D9891	2mg/mL	2ug/mL
**Neuronal Culture Media**				
BrainPhys Neuronal medium	STEMCELL Technologies	05790	N/A	N/A
B27 Supplement	Gibco	17504044	50X	1X
BDNF	PeproTech	450-02	10ug/mL	10ng/mL
NT-3	PeproTech	450-03	10ug/mL	10ng/mL
Laminin	Gibco	23017015	1mg/mL	1ug/mL
Poly-D-Lysine	Sigma Aldrich	A-003-E	1mg/mL	10ug/mL

Table S5. siRNA

Target	Manufacturer	Catalog/siRNA ID #
Control	Thermo Fisher	CAT# 4390843
SRRM2	Thermo Fisher	s24003
MSUT2	Millipore	EHU149521
PNN (1)	Thermo Fisher	s10758
PNN (2)	Thermo Fisher	s10759

Supplemental Figure 1: Polyserine-containing proteins enrich in tau aggregates

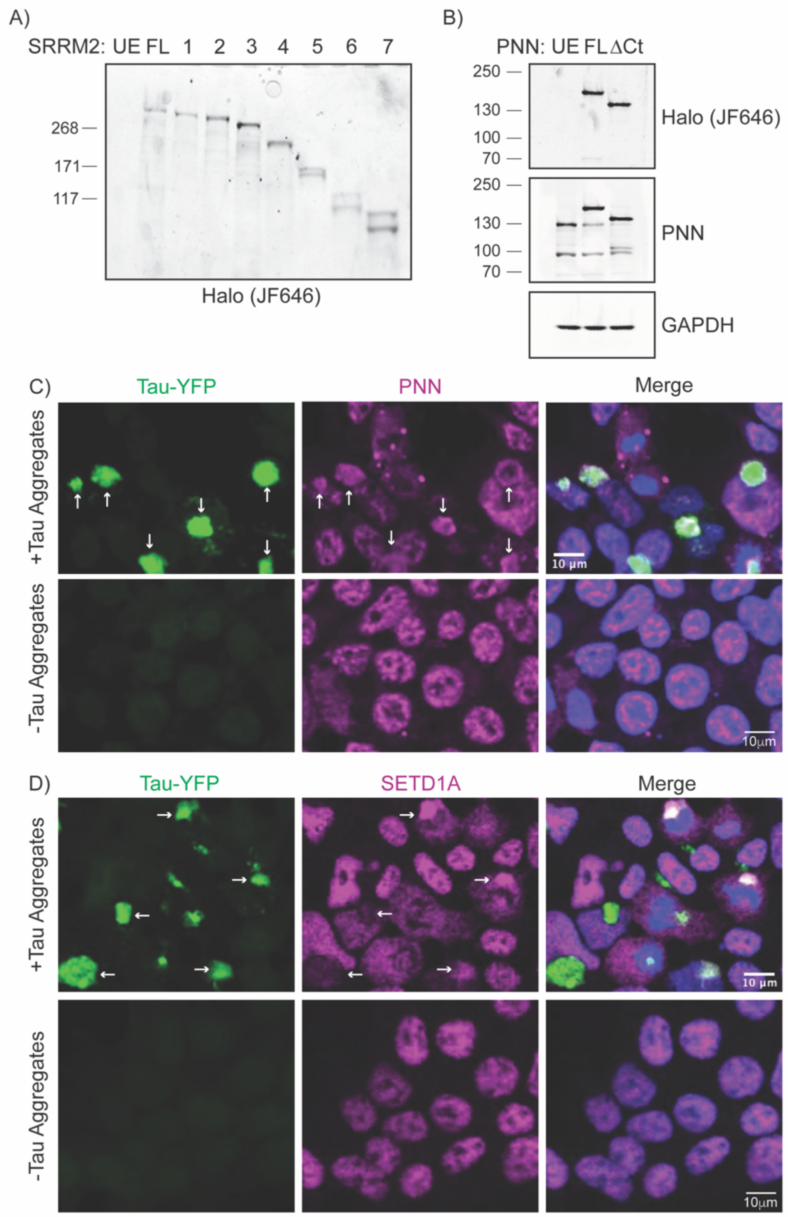

Figure S1: Polyserine-containing proteins enrich in tau aggregates
(A) Fluorescent imaging of Halo-tagged proteins from cell extracts of each SRRM2 truncation cell line. JF646 Halo ligand was added to cells prior to lysis and denaturation. **(B)** Fluorescent imaging of JF646 conjugated Halo-tagged proteins and Western blot for PNN and GAPDH from cell extracts of unedited, full-length (PNN_FL) and C-terminal truncated (PNN_ΔCt) cell lines. **(C)** Immunofluorescence of Tau-YFP (green), PNN (magenta), and DAPI (blue) in HEK293 biosensor cells with or without lipofection of clarified brain homogenate from tau transgenic mice (Tg2541). Tau aggregates colocalizing with PNN are denoted (white arrows). **(D)** Immunofluorescence of Tau-YFP (green), SETD1A (magenta), and DAPI (blue) in HEK293 biosensor cells with or without lipofection of clarified brain homogenate from tau transgenic mice (Tg2541). Tau aggregates colocalizing with PNN are denoted (white arrows).

Supplemental Figure 2: Characterization of Halo-tagged SRRM2 truncation cell lines

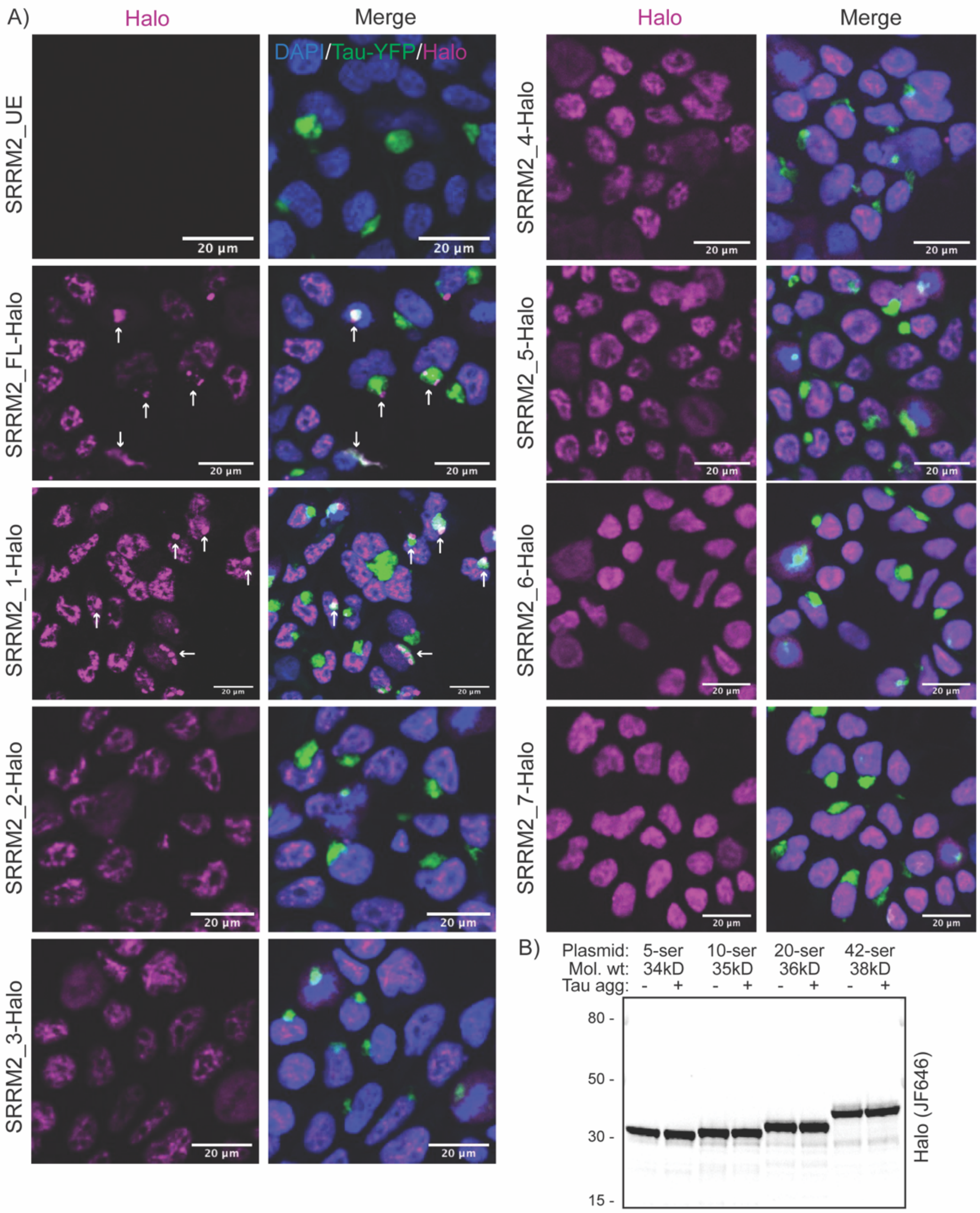

Figure S2: Characterization of Halo-tagged SRRM2 truncation cell lines
(A) Immunofluorescence of Halo (magenta), tau-YFP (green) and DAPI (blue) in Halo tagged SRRM2 truncation cell lines and SRRM2_UE (unedited, no Halo tag). White arrows show colocalization in SRRM2_FL-Halo and SRRM2_1-Halo. No colocalization observed in the other truncations. **(B)** Fluorescent imaging of an SDS-PAGE gel of Halo-tagged proteins in HEK293 cell lysate transfected with 5, 10, 20, or 42 polyserine-Halo constructs with or without transfection of clarified tau brain homogenate.

Supplemental Figure 3: SRRM2 and PNN cytoplasmic granules are sites of tau aggregation

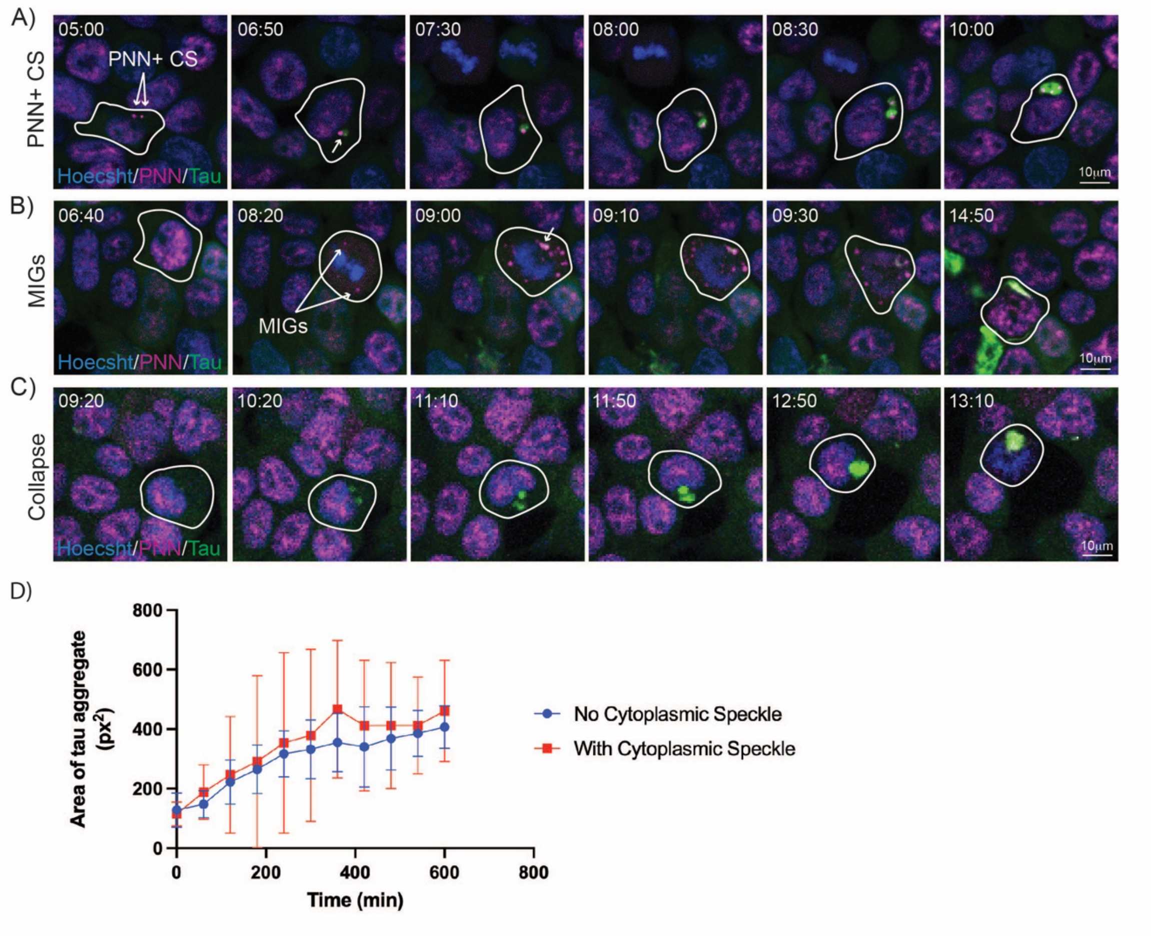

Figure S3: SRRM2 and PNN cytoplasmic granules are sites of tau aggregation
(A-C) Live imaging of Hoechst (blue), Tau-YFP (green) and PNN_FL-Halo (magenta) in HEK293 tau biosensor cells seeded with tau aggregates and monitored for 24 hours in 10-minute increments. Stills from live imaging display tau aggregate formation at PNN+ cytoplasmic speckles (A) (Movie 6), mitotic interchromatin granules (B) (Movie 7) and aggregate formation followed by nuclear collapse (C) (Movie 8). Time since the onset of imaging is displayed. **(D)** Comparison of tau aggregate growth over time between tau aggregates that initiated independently of cytoplasmic speckles or in close proximity to SRRM2+ speckles (<2μM). Four aggregates are quantified per condition and area is measured in pixels squared starting from the initial observance of the aggregate and continuing for 10 hours. Mean and standard deviation are shown.

Supplemental Figure 4: Stress granules are not preferred sites of tau aggregate formation

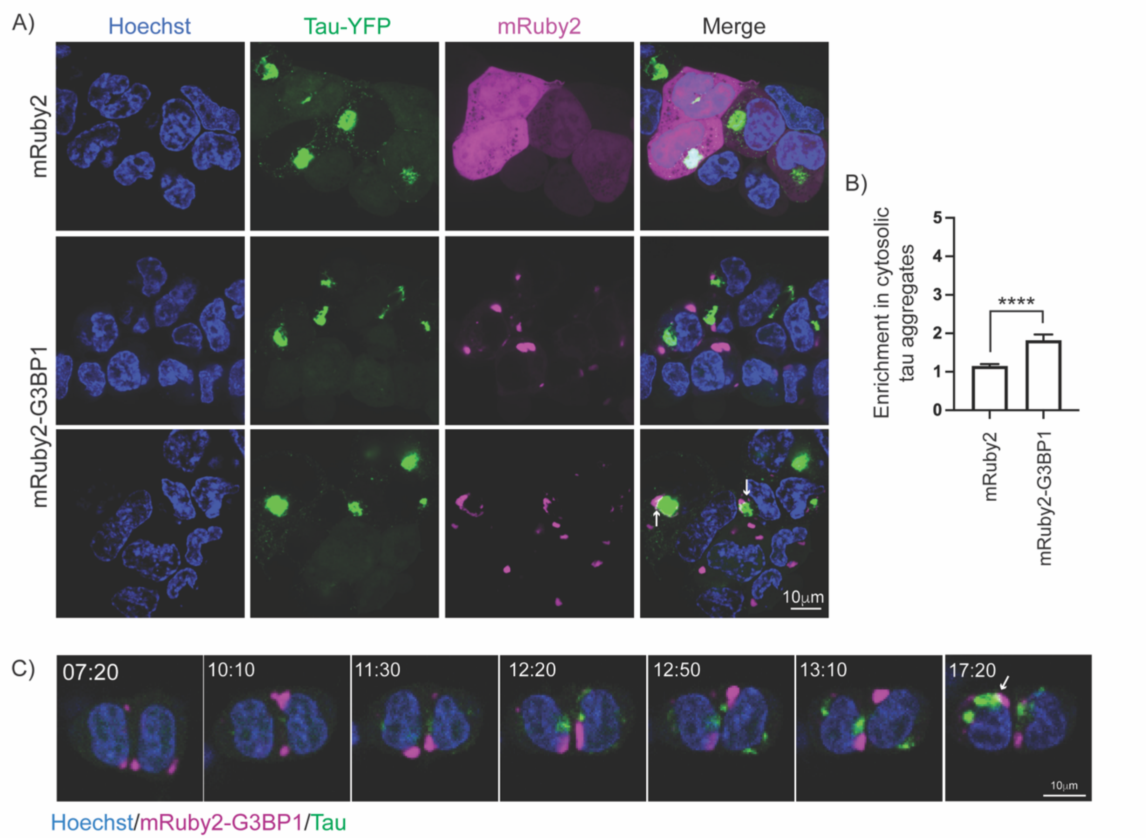

Figure S4: Stress granules are not preferred sites of tau aggregate formation
(A) Immunofluorescence of Hoechst (blue), Tau-YFP (green) and mRuby2 (magenta) in HEK293 tau biosensor cells stably transduced to express mRuby2 or mRuby2-G3BP1 30 hours post tau seeding and 24 hours after treatment with 50nM PatA. Docking of stress granules and tau aggregates are denoted by white arrows. **(B)** Quantification of the fold enrichment of mRuby2 in tau aggregates in HEK293 tau biosensor cells as in (A). Data represent mean and 95% CI. n>503 cells per group from three biological replicates. Statistics performed with Mann-Whitney test. (****) $P < 0.0001$. **(C)** Live imaging of Hoechst (blue), Tau-YFP (green) and mRuby2 (magenta) in HEK293 tau biosensor cells as in (A). Formation of stress granules and tau aggregates are independent with transient docking (white arrows) observed at later timepoints.

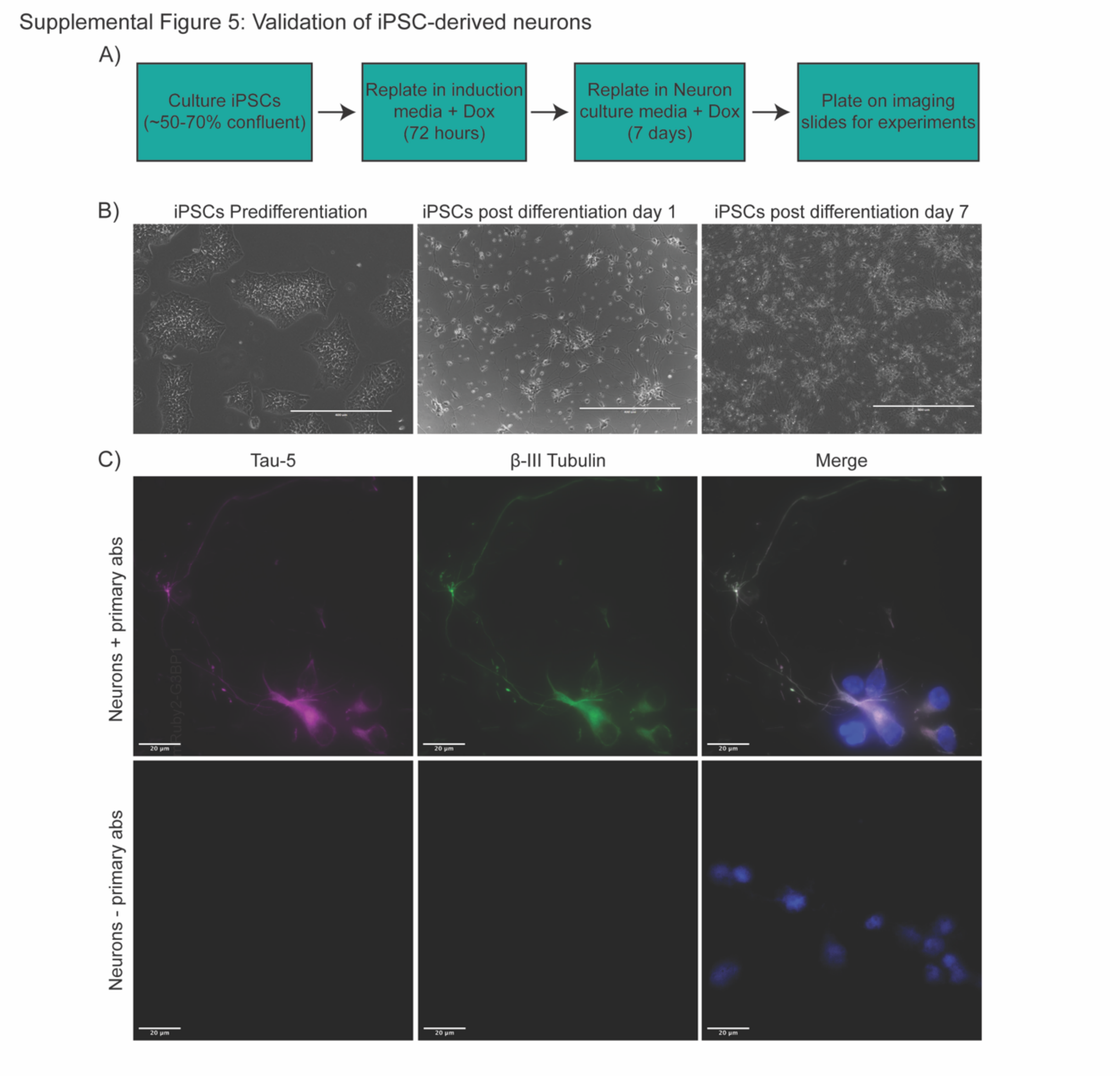

Figure S5: Characterization and validation of iPSC-derived neurons
(A) Differentiation protocol schematic for iPSC-derived iNeurons. **(B)** Brightfield images of iPSCs pre-differentiation, at day 1 post-differentiation, and day 7 post-differentiation. **(C)** Immunofluorescence of neuronal markers β-III Tubulin (green) and Tau-5 (magenta) and DAPI (blue) in iNeurons at 8 days post differentiation.

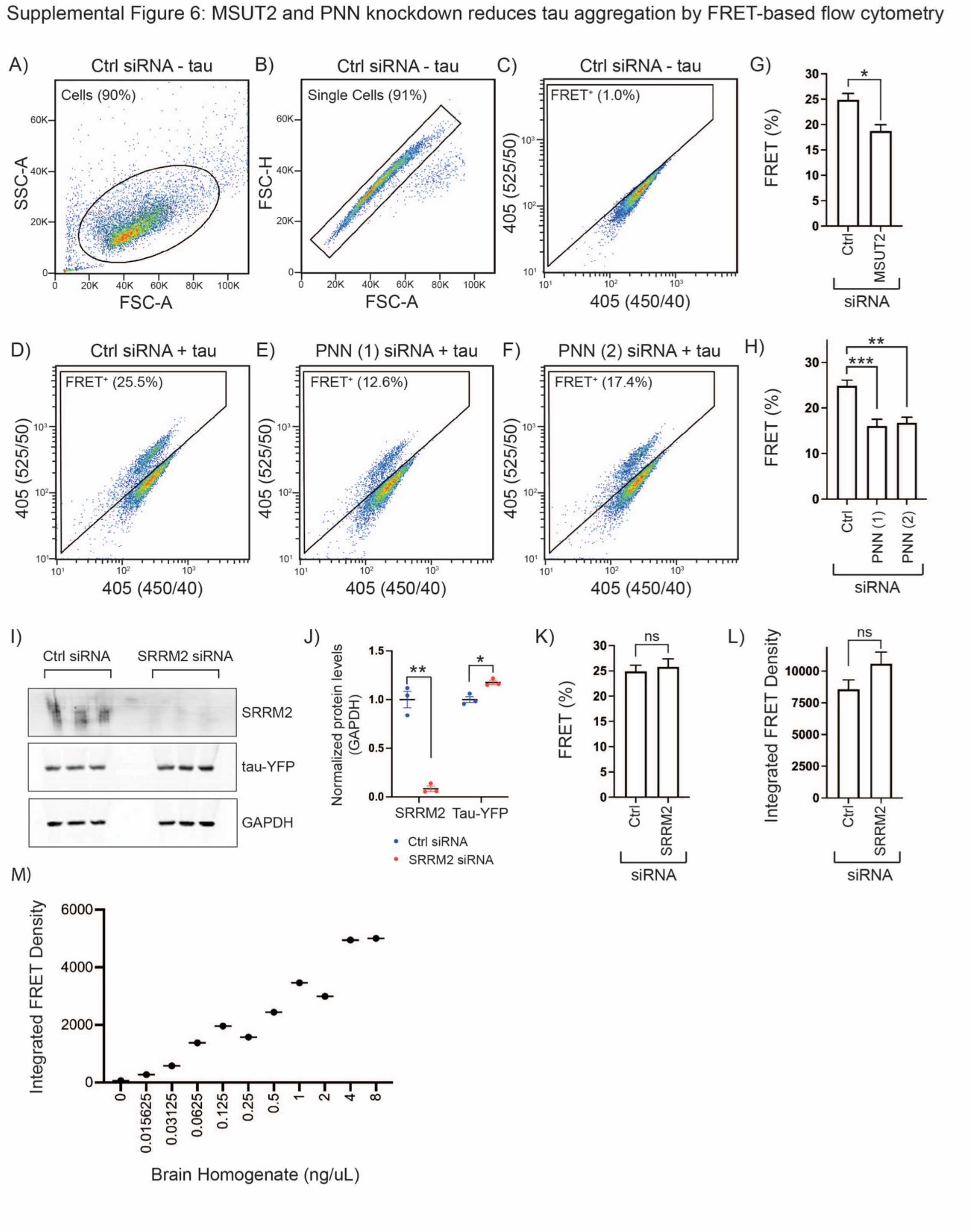

Figure S6: MSUT2 and PNN knockdown reduces tau aggregation by FRET-based flow cytometry **(A)** Plot of forward scatter area (FSC-A) versus side scatter area (SSC-A) with representative gating for HEK293 biosensor cells treated with control siRNA. **(B)** Scatter plot of FSC-A versus forward scatter height (FSC-H) for cells selected in (A) with representative gating for single cells of HEK293 biosensor cells treated with control siRNA. **(C)** Scatter plot of CFP [405 (450/40)] versus FRET [405 (525/50)] signal in HEK293 biosensor cells treated with control siRNA selected in (B) with representative gating for FRET+ cells**. (D-F)** Scatter plot of CFP [405 (450/40)] versus FRET

[405 (525/50)] signal in HEK293 biosensor cells treated with control or PNN siRNAs and seeded with clarified tau brain homogenate from populations as selected in (B) with representative gating for FRET+ cells. **(G)** Percentage of FRET+ HEK293 biosensor cells treated with control or MSUT2 siRNA and analyzed as in (Figure S6A-C). Data represent mean and SEM. Statistics performed with Mann-Whitney test. (*) $P < 0.05$. **(H)** Percentage of FRET+ HEK293 biosensor cells treated with control of each PNN siRNA measured by flow cytometry and analyzed as in (A-F). Data represent mean and SEM. Statistics performed with one-way ANOVA. (**) $P < 0.01$; (***) $P < 0.001$. **(I)** Western blot of SRRM2, tau-YFP and GAPDH protein levels in HEK293 biosensor cells treated with control or SRRM2 siRNA. **(J)** Quantification of Western blot shown in (I) normalized to GAPDH. Bars represent mean and SEM. Statistics performed with unpaired t-test with Welch's correction. **(K)** Percentage of FRET+ HEK293 biosensor cells treated with control or SRRM2 siRNA measured by flow cytometry and analyzed as in (Figure S6A-C). Data represent mean and SEM. Statistics performed with Mann-Whitney test. (ns) $P > 0.05$. **(L)** Integrated FRET Density of HEK293 biosensor cells treated with control or SRRM2 siRNA measured by flow cytometry and analyzed as in (Figure S6A-C). Data represent mean and SEM. Statistics were performed with Mann-Whitney test. (ns) $P > 0.05$. **(M)** Dose-response of integrated FRET density in following seeding with increasing concentrations of brain homogenate.

Supplemental Figure 7: Overexpression of polyserine does not alter tau levels

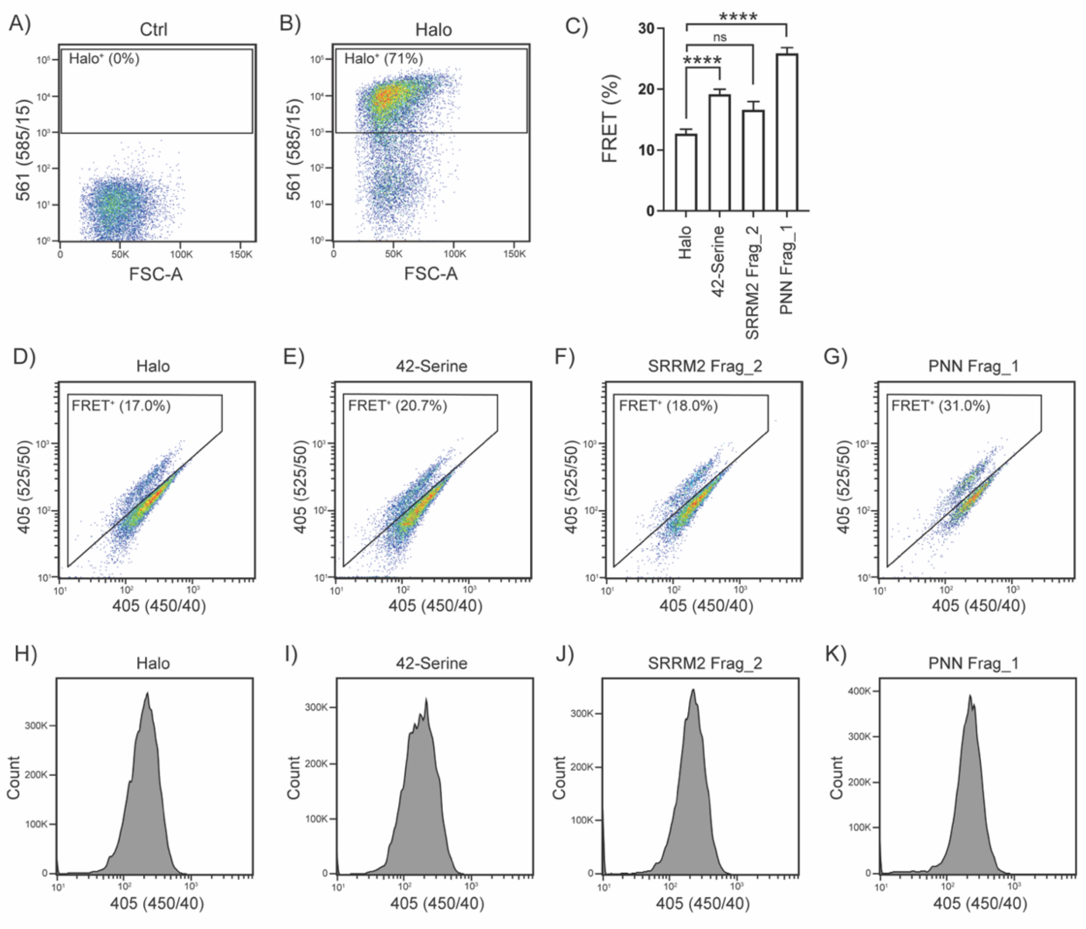

Figure S7: Overexpression of polyserine does not alter tau levels
(A, B) Scatter plot of FSC-A versus Halo intensity signal [561 (585/15)] in HEK293 biosensor cells mock transfected or transfected with Halo showing representative gating for Halo+ cells performed following gating steps detailed in Figure S6A,B. **(C)** Percentage of FRET+ HEK293 biosensor cells transfected with Halo, 42-Serine, SRRM2 Frag_2 and PNN Frag_1 constructs by flow cytometry and analyzed by gating in Figure S6A,B, subsequently Figure S7A,B and lastly for FRET positivity as in Figure S6C. Data represent mean and SEM. Statistics performed with one-way ANOVA. (ns) $P > 0.05$; (****) $P < 0.0001$. **(D-G)** Scatter plots of CFP [405 (450/40)] versus FRET [405 (525/50)] signal in HEK293 biosensor cells transfected with Halo, 42-Serine, SRRM2 Frag_2 and PNN Frag_1 after gating for cells and single cells as demonstrated in Figure 6A-B with representative gating for FRET+ cells. **(H-K)** Histograms of CFP [405 (450/40)] signal in HEK293 biosensor cells transfected with Halo, 42-Serine, SRRM2 Frag_2 and PNN Frag_1 after gating for cells and single cells.

Movie S1 (separate file). Movie showing tau aggregate (tau-YFP, green) nucleation from SRRM2+ MIGs (SRRM2-Halo, red).

Movie S2 (separate file). Movie showing a stable SRRM2+ CS (SRRM2-Halo, red) in the cytoplasm of a HEK293 cell independent of cell division.

Movie S3 (separate file). Movie showing tau aggregate (tau-YFP, green) nucleation from an SRRM2+ CS (SRRM2-Halo, red).

Movie S4 (separate file). Movie showing tau aggregate (tau-YFP, green) nucleation from SRRM2+ MIGs (SRRM2-Halo, red).

Movie S5 (separate file). Movie showing cell collapse where SRRM2-Halo (red) quickly merges with a tau aggregate (tau-YFP, green).

Movie S6 (separate file). Movie showing tau aggregate (tau-YFP, green) nucleation from PNN+ CS (PNN-Halo, red).

Movie S7 (separate file). Movie showing tau aggregate (tau-YFP, green) nucleation from PNN+ MIG (PNN-Halo, red).

Movie S8 (separate file). Movie showing cell collapse where PNN-Halo (red) quickly merges with a tau aggregate (tau-YFP, green).

Movie S9 (separate file). Movie showing tau aggregates (tau-YFP, green) forming independently of G3BP1-Halo (red)

Movie S10 (separate file). Movie showing tau aggregates (tau-YFP, green) forming independently of G3BP1-Halo (red). Example 2

Movie S11 (separate file). Movie showing tau aggregates (tau-YFP, green) nucleating from SRRM2ct-Halo (red) MIGs.

Movie S12 (separate file). Movie showing tau aggregates (tau-YFP, green) nucleating from 42serine-Halo (red) CSs.
